# Analysis of 182 cerebral palsy transcriptomes points to dysregulation of trophic signalling pathways and overlap with autism

**DOI:** 10.1038/s41398-018-0136-4

**Published:** 2018-04-23

**Authors:** Clare L. van Eyk, Mark A. Corbett, Alison Gardner, Bregje W. van Bon, Jessica L. Broadbent, Kelly Harper, Alastair H. MacLennan, Jozef Gecz

**Affiliations:** 1 Adelaide Medical School, Faculty of Health and Medical Sciences, University of Adelaide, Adelaide, SA Australia; 2 Robinson Research Institute, Faculty of Health and Medical Sciences, University of Adelaide, Adelaide, SA Australia; 3 Department of Human Genetics, Radboud University Medical Center, Nijmegen, The Netherlands; 4 South Australian Health and Medical Research Institute, Adelaide, SA Australia

## Abstract

Cerebral palsy (CP) is the most common motor disability of childhood. It is characterised by permanent, non-progressive but not unchanging problems with movement, posture and motor function, with a highly heterogeneous clinical spectrum and frequent neurodevelopmental comorbidities. The aetiology of CP is poorly understood, despite recent reports of a genetic contribution in some cases. Here we demonstrate transcriptional dysregulation of trophic signalling pathways in patient-derived cell lines from an unselected cohort of 182 CP-affected individuals using both differential expression analysis and weighted gene co-expression network analysis (WGCNA). We also show that genes differentially expressed in CP, as well as network modules significantly correlated with CP status, are enriched for genes associated with ASD. Combining transcriptome and whole exome sequencing (WES) data for this CP cohort likely resolves an additional 5% of cases separated to the 14% we have previously reported as resolved by WES. Collectively, these results support a convergent molecular abnormality in CP and ASD.

## Introduction

With a frequency of around 2 per 1000 live births, cerebral palsy (CP) is the most common motor disability of childhood^1,2^. It is the result of a non-progressive interference, lesion or abnormality in the developing brain, occurring in the antenatal, perinatal or early post-natal period, and is often accompanied by additional features including intellectual disability (ID), autism spectrum disorder (ASD), epilepsy and visual and hearing impairment. The overall clinical spectrum of CP is highly heterogeneous, encompassing multiple clinical types, multiple patterns of neuropathology on brain imaging and multiple associated developmental pathologies.

A number of clinical risk factors have been described for CP, including very pre-term delivery, placental pathology, intrauterine exposure to infection, intrauterine growth restriction (IUGR), breech presentation, bleeding during pregnancy and multiple pregnancy^3^. These factors suggest that CP is frequently the result of long-standing intrauterine pathology and not a single event during late labour or birth. For most cases of CP, the aetiology of the brain injury is not well understood, however recent reports suggest a significant genetic contribution^4–17^. In this study, we aimed to elucidate gene networks and pathways contributing to CP, as well as to assist prioritisation of genetic variants, by examining transcriptomes of a cohort of 182 clinically heterogeneous CP cases, all of which have previously been analysed by whole-exome sequencing^5^ (Supplementary Table S1).

## Materials and methods

### Samples

Epstein-Barr virus immortalised B-cell lines (LCLs) were established from peripheral blood lymphocytes of patients and controls. Lymphoblastoid cell lines for 182 CP cases are from the Australian Collaborative Cerebral Palsy Research Group Cerebral Palsy Biobank and were derived at Genetic Repositories Australia (Sydney, Australia), an Enabling Facility supported by NHMRC Grant 401184. Additionally, we utilised 20 unaffected controls from the Neurogenetics Research Group collection (University of Adelaide, Australia). Samples from the gEUVADIS data set are originally from Coriell Cell Repositories. Additional control cell lines for quantitative real-time PCR were obtained from the Genetic Repositories Australia ‘Aussie Normals Collection’. Cell lines are routinely tested for mycoplasma contamination. This study was approved by the Women’s and Children’s Health Network (WCHN) Human Research Ethics Committee (reference number: HREC/15/WCH/148). Written consent was given, either by the participant or their guardian, for the use of their sample in CP research.

### Cell culture

Once established, LCLs were cultured in RPMI 1640 (Sigma) supplemented with 10% foetal calf sera, 2 mM l-Glutamine, 0.017 mg ml^−1^ benzylpenicillin and grown at 37 °C with 5% CO_2_.

### RNA preparation

For RNA sequencing, RNA was extracted from CP cell pellets and in-house controls using the RNeasy mini-kit (Qiagen) according to manufacturer’s instructions. RNA concentration was assessed by Nanodrop and RNA quality was measured using an Agilent Bioanalyser. All RNA samples used for RNA sequencing had RIN ≥8.9. RNA from CP cell lines and GRA ‘Aussie Normals Collection’ cell lines for quantitative reverse transcription PCR (qRT-PCR) validation were extracted using Trizol (Invitrogen) followed by RNeasy mini-kit (Qiagen) according to manufacturers’ instructions.

### RNA-seq

Libraries were prepared using the TruSeq v2 kit (Illumina) to construct unstranded libraries with a mean fragment size of 150 bp. Libraries underwent 50-bp paired-end sequencing on an Illumina HiSeq 2500. RNA-seq reads were aligned to the hg19 build of the reference genome and a pre-built splice junction database generated from known gene models (UCSC genes) using Tophat^18^. Counts for each transcript were determined using HT-Seq^19^ and statistical analysis performed using the EdgeR package in R^20^.

### Outlier analysis

For outlier-gene analysis, we calculated the *Z* statistic for each gene by using the ‘scale’ function in R. Mean and SD were calculated for each expressed gene in cases and controls separately. We selected a cutoff to define whether a gene was an outlier in cases or controls. In this analysis, an outlier was defined as a gene with expression in a sample at least 2 SD from the mean expression of all samples where a likely dysregulating genetic variant was detected in the gene, or 4 SD from the mean expression where there was no genetic variation detected in the gene by WES.

### Validation sequencing

Additional genetic variants from WES^5^were prioritised following outlier analysis. Variants were validated by Sanger sequencing using BigDye terminator chemistry 3.1 (ABI) and analysed using a 3730xl genetic analyzer (Applied Biosystems, Foster City, CA, USA). Sequencing data was analysed using DNASTAR Lasergene 10 Seqman Pro8 (DNASTAR, Inc. Madison, WI, USA). Validations were performed using genomic DNA isolated from whole blood where possible or alternatively, DNA extracted from LCLs. Where possible, segregation in patient–parent trios was performed to confirm the inheritance pattern of the variants.

### Differential expression analysis

Prior to differential expression (DE) analysis, data were normalised using the ComBat function from the SVA package in R^21^. Factors accounted for were batch and gender. Following ComBat normalisation, data were log2-transformed and DE was assessed by a linear regression method using the limma package in R. For each coefficient in the linear model, empirical Bayes-moderated *t*-statistics and their associated *p* values were used to assess the significance of the observed expression changes. Since we were unable to account for age-specific gene expression in samples from the gEUVADIS data set, we also analysed our CP cohort and in-house controls separately, applying linear regression of expression values against age and gender as part of our model. We identified 387 differentially expressed genes which were significant in both analyses when we applied a significance threshold of log-fold change > ±0.5 and corrected *p* value <0.001 for analysis including gEUVADIS controls and *p* value <0.05 for analysis with in-house controls regressed for age and gender.

### Gene ontology analysis

Gene ontology (GO) enrichment for differentially expressed genes was performed using PANTHER statistical over-representation test (http://www.pantherdb.org/) with Bonferroni correction^22^.

### Ingenuity pathway analysis

Gene network analysis was performed using ingenuity pathway analysis (IPA; Qiagen). Gene lists were imported and analysed using the ‘core analysis’ option to perform expression analysis. Standard settings were used with both direct and indirect interactions between molecules considered and only experimentally observed interactions included. For networks limited to central nervous system (CNS), tissues and cell lines were limited to nervous system tissues and primary cells, and CNS cell lines.

### qRT-PCR

We pooled 0.5 μg of total RNA from each of the five independent samples, giving a total of 2.5 μg of total RNA in each validation pool. For each validation pool, we then made complementary DNA using SuperScript III (Invitrogen) and random hexamers according to manufacturer’s instructions. We performed qRT-PCR on a StepOnePlus real-time PCR system (Applied Biosystems, Foster City, CA, USA) using pre-designed Taqman gene expression assays and Taqman gene expression master mix (Applied Biosystems). Assay numbers used were Hs00998100_m1 (ACTN1), Hs00915142_m1 (FGFR1), Hs00171191 (FBN1), Hs01102156_m1 (KLHL14), Hs00183378_m1 (RASGRP2), Hs00249930_s1 (RBMS1), Hs01398501_m1 (KDM7A), Hs00325999_m1 (TET2), Hs00241801_m1 (ARHGAP6), Hs01026795_m1 (TUBA8), Hs00153462_m1 (LMNA), Hs00286908_m1 (KIF21A) and Hs00602051_mH (FSCN1). Relative expression levels were determined using a standard curve and values normalised to the quantity of Actin-B (catalogue #4326315E) in duplexed reactions.

### Gene lists for other disorders

Lists of genes associated with other neurodevelopmental and movement disorders are from Nijmegen genome diagnostics (http://www.genomediagnosticsnijmegen.nl/index.php/en/) for ID, epilepsy and movement disorders, from the Simons Foundation Autism Research Initiative (SFARI) database for Autism (https://gene.sfari.org/autdb/HG_Home.do) and from the Schizophrenia database (SZDB) for Schizophrenia (http://www.szdb.org/). Statistical significance and representation factor (number of overlapping genes over expected number of overlapping genes) of the overlap between each neurodevelopmental/movement disorder gene list and genes differentially expressed in CP or genes within network modules of interest was calculated using a hypergeometric probability test (http://nemates.org/MA/progs/overlap_stats.html), where the whole-gene population was defined as the 9884 genes robustly expressed in LCLs.

### WGCNA

Unsigned co-expression networks were built using the WGCNA package in R^23^. A total of 9881 genes were included in the network following filtering with the goodSamplesGenes function in WGCNA to remove genes with too many missing values. Network construction for the whole data set was performed using the blockwiseModules function. Using this function, a pairwise correlation matrix was computed for each set of genes and an adjacency matrix was calculated by raising the correlation matrix to a power of 6, as recommended for unsigned co-expression networks^23^. Using this approach, we built networks using control samples only, all samples and CP samples only. For each pair of genes, topological overlap measure was then calculated based on the adjacency matrix to give a robust measure of network interconnectedness. The topological overlap dissimilarity was then used as input for average linkage hierarchical clustering and modules were defined as branches of the resulting clustering tree using the hybrid dynamic tree-cutting function. We used a minimum module size of 40 genes with a minimum module merging height of 0.1. We merged modules using the moduleMergeUsingKME function with parameters threshPercent = 50, mergePercent = 25, reassignScale = 0.6 to generate our final network of 13 modules. We then summarised each module in the network by a module eigengene value, which is the principal component of the standardised module expression profile. Module membership (module eigengene connectivity, kME) was defined as the correlation between gene expression values and the module eigengene. Genes were assigned to a module if they had a high-module membership (kME >0.7), allowing them to belong to more than one module. Genes that did not fulfil this criteria for any module were assigned to the grey module. We then assessed module preservation between the networks built using all data, control data only and CP cases only using the modulePreservation function. In all cases, good module preservation was observed (*Z*-summary >10), therefore we used the modules defined for the control network for the remainder of our analysis. We calculated module eigengene values for each of the control and CP samples with modules as defined for this control network. We then calculated module eigengene significance for each module in the network for clinical factors: CP status, age, sex, gestation, co-morbidity for other neurodevelopmental disorders, presence of known CP risk factor, maternal smoking, and identified genetic variant of interest. Module gene list enrichment analysis was performed using the userListEnrichment function with options useBrainLists and useBrainRegionMarkers^24^.

### Comparison to autism cortex network

To compare our network to the autism cortex network^24^, we reconstructed the network with the 5208 genes common to data from both studies. Common genes were found using the collapseRows function in WGCNA^25^ and the autism network was reconstructed using the parameters described in the original study. We then used the userListEnrichment function to identify modules in the autism cortex network enriched for genes from modules in our LCL network.

## Results

We analysed gene expression of patient-derived lymphoblastoid cell lines from the CP cohort, alongside 20 lymphoblastoid cell lines from individuals unaffected by CP (Table S2) using Illumina paired-end RNA sequencing. Analysis of gene expression changes was performed together with data from an additional 100 lymphoblastoid cell lines from the gEUVADIS RNA sequencing project for 1000 Genomes samples^26^ (Table S3). While lymphoblastoid cell lines are of peripheral blood origin and therefore cannot recapitulate all gene expression signatures of the affected tissue, they provide a valuable source of patient-derived material for gene function and biomarker analysis, with several studies demonstrating appreciable neuronal relevance^27–29^.

**Fig. 1:**
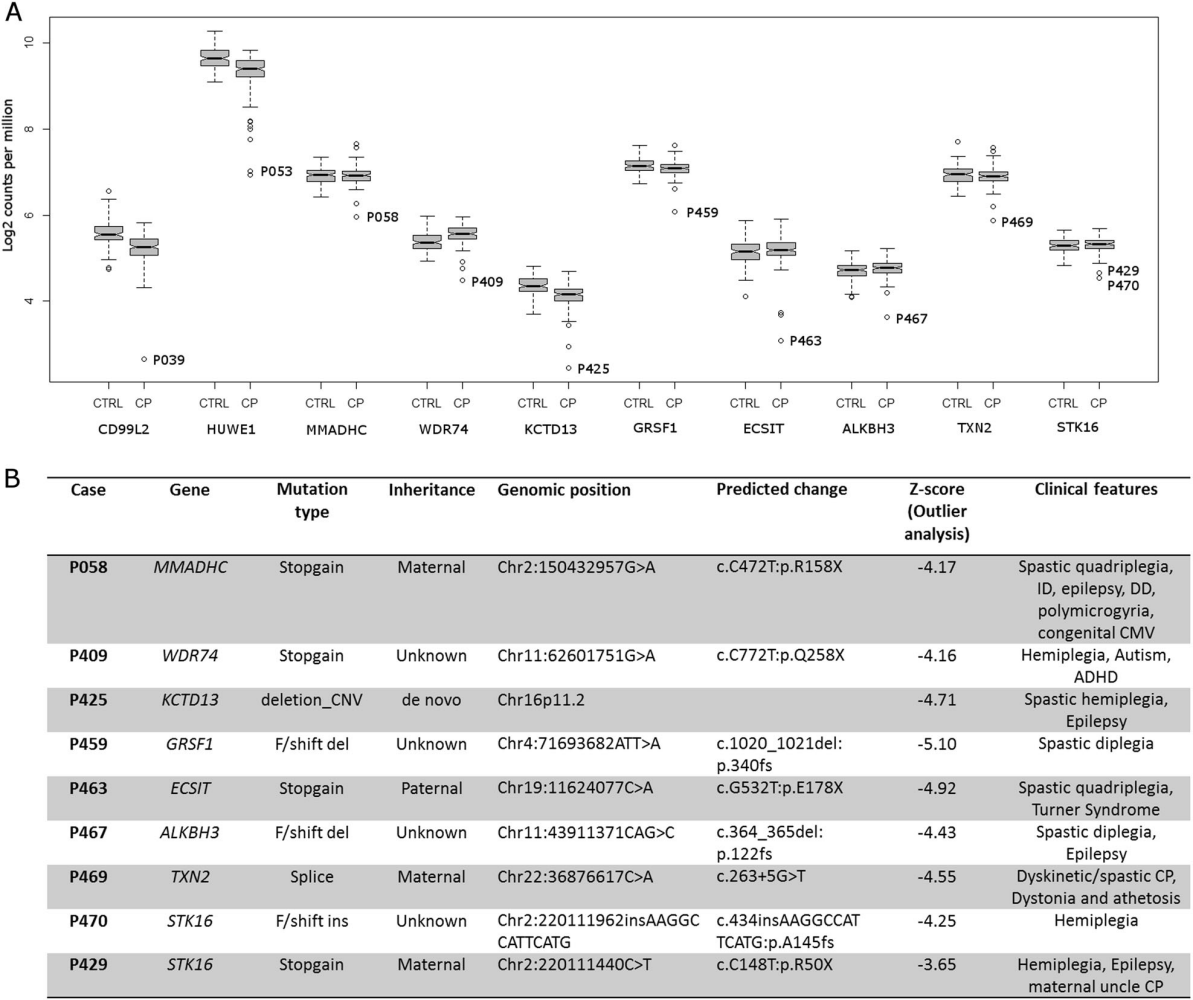
Outliers supported by genetic variants in select cerebral palsy cases. All cases have previously been analysed by WES. Samples had expression >4 SD from the mean. **a** Outliers supported by a genetic variant from WES study^5^. Variants were selected on the basis of known function and association with other human disorders, as well as predicted effect on gene function. See also Supplementary Table 1. **b** Additional genetic variants of interest identified in this study due to support by RNA-seq data. CNV copy-number variation, f/shift frameshift, del deletion, ins insertion

We first performed outlier analysis on all 182 CP cases to identify genes that are significantly downregulated or upregulated compared to expression in other cases in the CP cohort. We also examined the variation in expression of genes of interest in our CP cohort compared to controls (Fig. 1a) to determine whether the difference in gene expression was likely to be contributing to or causing CP. This approach provided support for a functional effect of several genetic variants previously identified in our WES study^5^, including a stop-gain mutation in *CD99L2* [MIM 300846] and a compound heterozygous mutation in *HUWE1* [MIM 300697] (Fig. 1a). In addition, outlier analysis provided support for a functional effect of a number of additional genetic variants, which were not prioritised in the WES study (Fig. 1a, b), partly since this study focussed on the 98 CP cases where both parents were available^5^. We also identified a number of outlier genes of potential interest where no genetic variant was identified by WES (Figure S1). For these genes, the underlying genetic variant may have been missed by WES, the variant may reside in a non-coding region, or the gene expression change may be a downstream effect of other genetic or environmental factors. Several such outlier genes identified have been associated with other neurodevelopmental disorders, including *CHD8*^30^ [MIM 610528], *KIDINS220*^31,32^ [MIM 615759], *DIP2B*^33^ [MIM 611379] and *TBL1XR1*^30,34,35^ [MIM 608628]. Conservatively, considering only the expression outlier genes where a deletion, stop-gain or frameshift variant was detected (Fig. 1a, b), we may have resolved another nine (5%) CP cases from the cohort (*n* = 182).

**Fig. 2:**
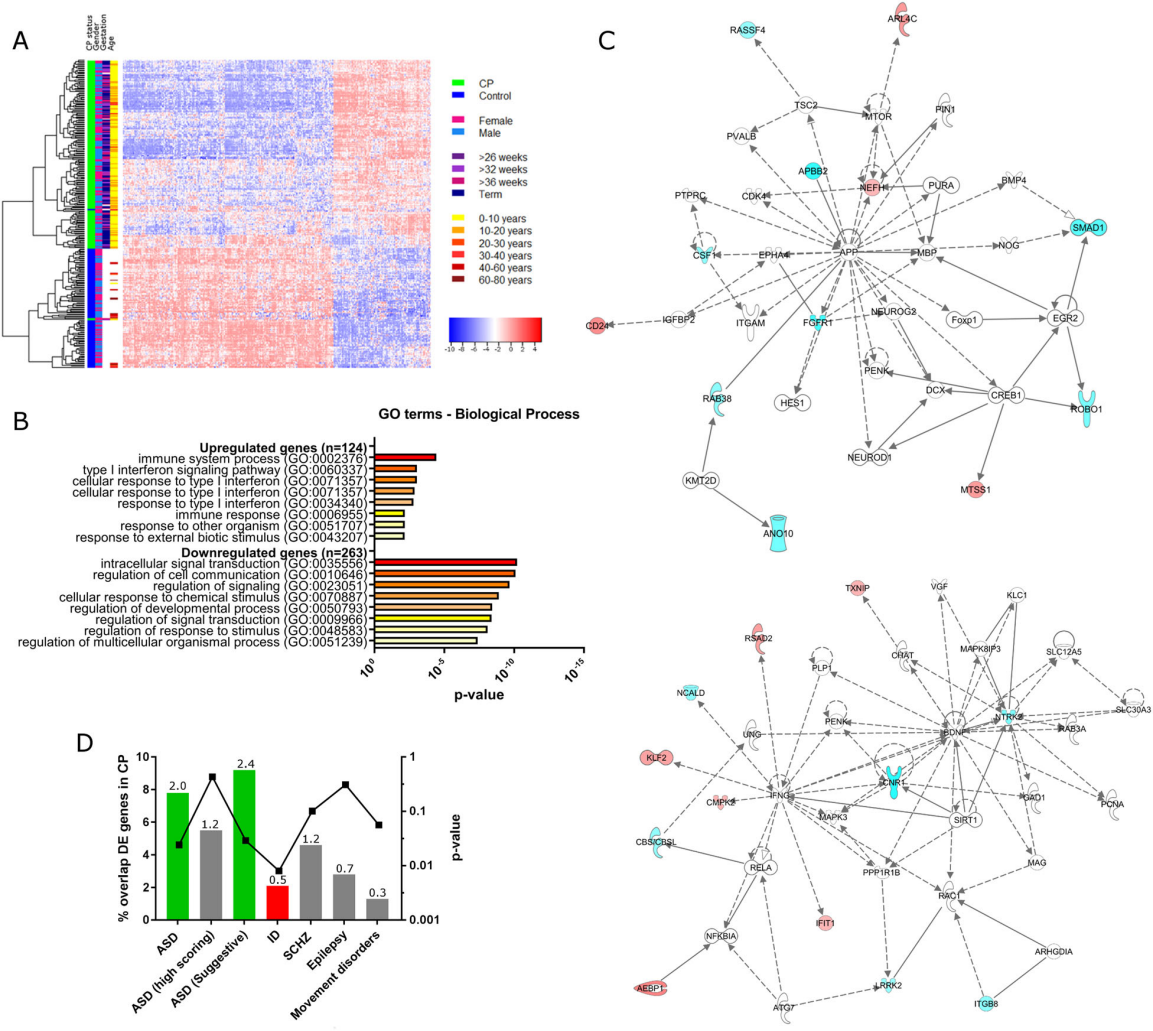
Differential expression analysis of CP cases compared to controls. **a** Unsupervised hierarchical clustering of CP cases and controls based on differentially expressed transcripts (*n* = 387, logFC >±0.5, Bonferroni-corrected *p* value <0.001). **b** Gene Ontology term enrichment for differentially expressed genes in CP cases compared to control cases. Bonferroni-corrected *p* values are shown. **c** Top two networks identified by ingenuity pathway analysis (right-tailed Fisher’s exact test, *p* = 1 × 10^−18^ where *p* is the probability of finding *f* or more hub genes from the network in a set of *n* randomly selected genes). Input was the top differentially expressed gene list (logFC >±1.2, Bonferroni-corrected *p* value <0.001) for CP cases compared to controls, with connections limited to those with evidence from CNS cell lines. Highlighted genes are those differentially expressed in CP cases compared to controls, with blue denoting downregulation and red denoting upregulation. Solid lines represent direct interactions, while dashed lines represent indirect interactions. **d** Overlap between differentially expressed genes in this study and genes associated with neurodevelopmental and movement disorders. Gene lists were compiled from publically available databases (see Materials and methods). Left axis is bar plot of percentage overlap between each gene list and CP DE gene list, with numbers above each bar being the fold difference of the observed to expected overlap between these gene lists. Right axis is a dot plot of *p* values derived from hypergeometric distribution analysis of overlap between each gene list and the genes differentially expressed in CP. A significant over-representation of differentially expressed CP genes was seen in the ASD gene list, and a significant under-representation of genes associated with ID

For DE analysis, we used a stringent filter for robust expression (more than five counts per million in at least 120 samples), before removing batch effects in the data and adjusting for gender as a covariate using the ComBat function^21^ (Fig. 2). In-house controls and gEUVADIS controls were grouped together in multidimensional scaling (MDS), (Figure S2D), suggesting that the batch effect had been successfully removed. We then performed DE analysis on the ComBat-normalised data using the EdgeR package in R^20^ (Table S4). To account for expression changes resulting from differences in age of our CP and control cohorts, we also performed differential expression analysis on data for all CP samples compared to our 20 in-house control samples. Data were normalised for library size and DE analysis was performed using a linear model with age and gender as covariates. We identified 387 genes that showed significantly altered expression in both analyses (logFC >±0.5, Bonferroni-corrected *p* value <0.001 for batch-corrected data including gEUVADIS and in-house controls and *p* < 0.05 for data including only in-house controls), with 124 genes upregulated and 263 genes downregulated (Table S5, Figure S3). We validated a cross-section of the differentially expressed genes by quantitative real-time PCR with an independent set of 10 control cell lines (Table S6), confirming RNA-seq differential expression in 83% (10/12) of the genes tested (Figure S4).

Unsupervised hierarchical clustering based on the 387 genes differentially expressed between CP and control cases showed distinct clustering of the majority of CP samples (Fig. 2a) with no apparent clustering of samples based on potential confounding factors including age, gender, sex or gestational age. GO enrichment analysis using PANTHER^22^ showed that the 263 genes downregulated in CP cases are enriched for GO categories implicated in signal transduction and cell signalling, while the 124 upregulated genes are enriched for GO categories relating to immune response (Fig. 2b). We also used IPA software (Qiagen) to visualise key networks of genes and pathways over-represented in our list of differentially expressed genes. We generated a list of the top dysregulated genes (logFC >±1.2, Bonferroni-corrected *p* value <0.001, 82 genes). The top network identified by IPA was centred on the extracellular signal-regulated kinase 1/2 (ERK1/2) mitogen-activated protein kinase (MAPK) signalling pathway (right-tailed Fisher’s exact test, *p* = 1 × 10^−42^). Limiting connections to those derived from CNS cells revealed two top networks, the first centred on the amyloid precursor protein A (APP), with FGFR1 being a central downstream component of the pathway, and the second on brain-derived neurotrophic factor (BDNF) (right-tailed Fisher’s exact test, *p* = 1 × 10^−18^, representative pathway showing key connections between molecules in these gene networks shown in Fig. 2c). This finding is consistent with the enrichment for GO categories involving inflammation and signal transduction and cell signalling in this gene list. Both the BDNF receptor NRTK2 (tropomyosin-related kinase receptor type B, trkB) [MIM 600456] and fibroblast growth factor (FGF) receptor FGFR1 [MIM 136350] are downregulated in CP compared to controls (log-fold changes: *NTRK2 −*1.59328, Bonferroni-corrected *p* value = 6.88*E*−26, *FGFR1* −2.30493, Bonferroni-corrected *p* value = 2.92*E*−29 in CP cases compared to all controls), suggesting a broader dysregulation of trophic signalling pathways in CP. We also observed a significant downregulation of tet methylcytosine dioxygenase 2 (*TET2*) [MIM 612839], a factor known to regulate BDNF signalling via de-methylation of the BDNF promoter^36^ and upregulation of *KDM7A*, a histone demethylase that regulates FGF signalling and is important for neural differentiation^37^ (log-fold changes: *TET2* -0.62, Bonferroni-corrected *p* value = 1.97*E*−12, *KDM7A* 1.22, Bonferroni-corrected *p* value = 1.65*E*−26), implicating epigenetic changes in the molecular pathway leading to CP.

Using gene lists derived from public databases (Materials and methods), we examined our list of differentially expressed genes (logFC >±0.5, Bonferroni-corrected *p* value <0.001) for enrichment with genes associated with other neurodevelopmental or movement disorders. We found a modest but significant enrichment for genes associated with autism (fold difference = 2.0, *p* < 0.024) (Fig. 2d), but no significant enrichment for genes associated with schizophrenia, epilepsy or movement disorders, and a statistically significant under-representation of genes associated with ID (fold difference = 0.5, *p* < 0.008). Enrichment for autism genes was not significant for genes associated with syndromic forms of autism, or for those categorised as either high-confidence or strong candidate autism genes in the SFARI database, but was significant for genes categorised as having suggestive evidence for a role in autism (Fig. 2d, fold difference = 2.4, *p* < 0.029). Genes in this category are either supported by several lines of nominal evidence but lack functional evidence, or are supported by nominal evidence lacking genome-wide significance or consistent replication but meet at least one of four accessory criteria that include altered expression in autism samples, involvement in a related disorder or association with another autism risk gene via genetic epistasis. Therefore, genes falling into these categories are less likely to be large effect risk genes for autism and instead may be downstream markers of pathology or play broader roles in neurodevelopmental processes.

**Fig. 3:**
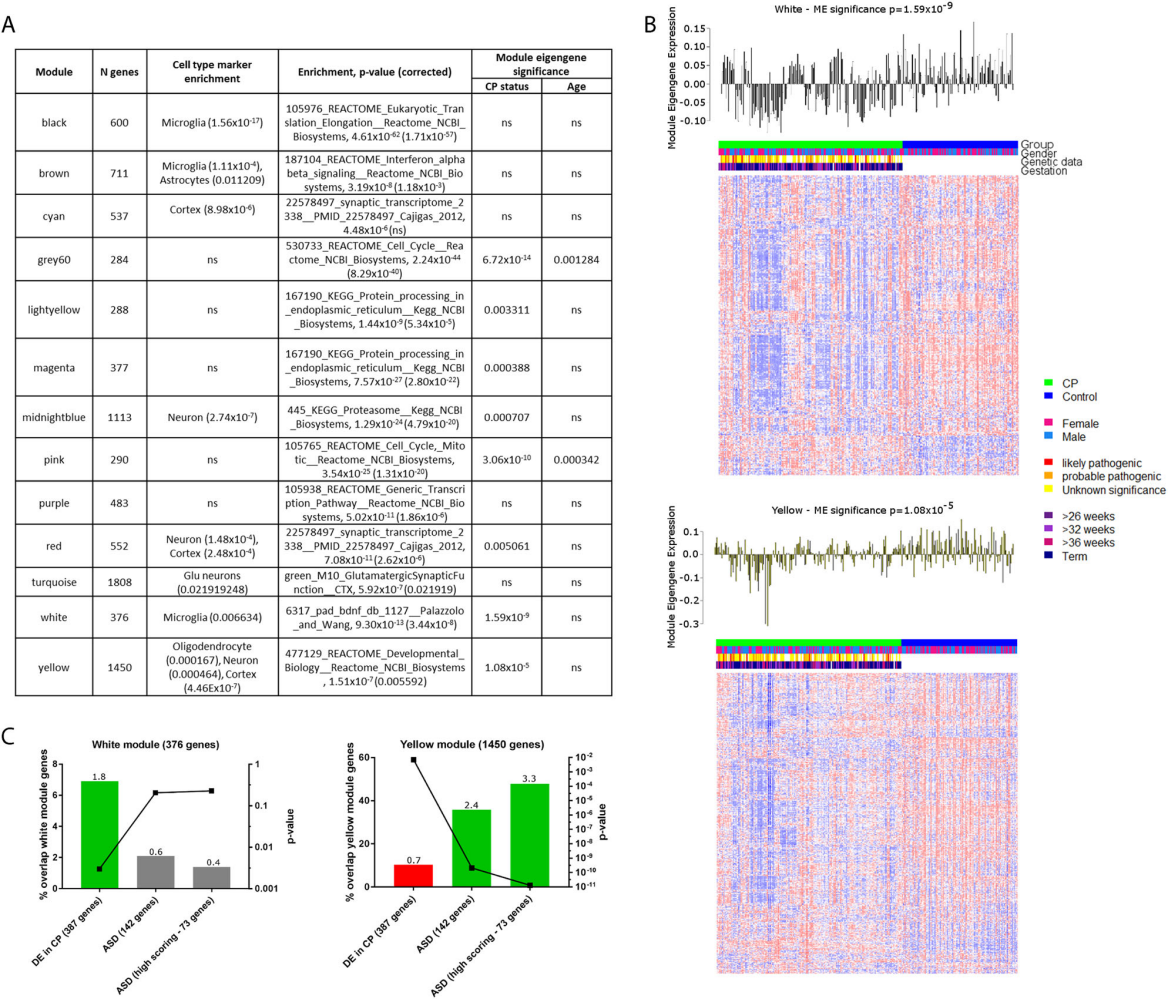
Gene co-expression modules in the control lymphoblastoid cell line network. **a** Modules in control network with cell type marker enrichments and functional annotation enrichment. Module eigengene significance for each module denotes whether there is an association between expression of the module and characteristics of the samples. **b** Heatmap of genes belonging to the white and yellow gene co-expression modules, which have the most significant association with CP status. All genes in the white module are shown (376 genes), while the larger yellow module consisting of 1450 genes was limited to the top 300 genes ranked by connectivity to all other genes in the module. Corresponding module eigengene values (*y*-axis) across samples (x-axis) (top). **c** Enrichment analysis (hypergeometric probability) of white and yellow modules with ASD gene sets and differentially expressed genes in CP. Left axis is bar plot of percentage overlap between each gene list and CP DE gene list, with numbers above each bar being the fold difference of the observed to expected overlap between these gene lists. Right axis is a dot plot of *p* values derived from hypergeometric distribution analysis of overlap between each gene list and the genes in the white and yellow modules

We next applied weighted gene co-expression network analysis (WGCNA), to examine differences in gene expression in a systems-based context. We constructed an unsigned co-expression network for all control samples (as described in^24^, Materials and methods) and examined preservation of the network structure in CP samples. All 13 control modules were highly conserved (*z*-summary ≥10) in the CP network, suggesting overall preservation of gene expression at a network level. We performed module eigengene analysis to summarise the expression levels of genes within each module as a module eigengene value (similar to a principal component score). In this analysis, a module eigengene value was calculated for each cell line for each module and standard parametric analyses (*t* tests or student asymptotic *p* values for correlation) was performed to determine which modules were correlated with CP status or other factors (age, gender, gestation, presence of neurodevelopmental comorbidities, presence of clinical risk factors associated with poor developmental outcome including prematurity, IUGR, pre-eclampsia and thyroid problems in pregnancy). No factor other than CP status was correlated with any module, except age, which was significantly correlated with two modules (grey60 and pink). The most significant correlation with CP status was seen for the white and yellow modules (Fig. 3a, Bonferroni-corrected *p* values 1.59 × 10^−9^, 1.08 × 10^−5^, respectively).

Cell type/cellular process enrichment analysis showed that the white module is enriched for markers of BDNF signalling (corrected *p* = 3.44 × 10^−8^) and also for microglial markers (corrected *p* = 0.006634), while the yellow module is enriched for genes involved in developmental processes (corrected *p* = 0.0056) and also for genes expressed in cortex (corrected *p* = 4.46 × 10^−7^). Both the white and yellow module eigengenes are under-expressed in CP cases compared to controls, suggesting that genes in these modules are downregulated in CP. While the white module is enriched for genes differentially expressed genes in CP (fold difference = 1.8, *p* < 0.003), the yellow module has fewer than expected genes differentially expressed in CP (fold difference 0.7, *p* < 0.007), and instead is enriched for high-confidence autism candidate genes (fold difference = 3.3, *p* < 1.28 × 10^−11^) (Fig. 3c). This finding mirrors transcriptional changes reported in ASD that show that differentially expressed genes and high-confidence, large effect size, autism risk genes lie in different co-expression networks^24,38^.

We next compared our network modules to those from the autism cortex network^24^. We reconstructed the autism unsigned co-expression network using the parameters as reported and looked for enrichment of our network modules with genes from the autism network modules (Table S8). Of the two network modules of interest for autism (M16 and M12), only the M16 module was preserved in our LCL network. We found marginal enrichment (not significant after correction for multiple testing) of the white module with genes from the M16 module from the autism cortex network (Voineagu_M16). The yellow module is significantly enriched for genes from the Voineagu_M16 module (corrected *p* = 6.48 × 10^−17^), however, it is more significantly enriched for two other modules, Voineagu_M10 and Voineagu_M13, both of which are enriched for astrocyte markers^24^. In a larger subsequent study, the signal from the Voineagu_M16 module was further honed to a smaller module, Gupta_M5, which was enriched for M2 microglial cell state and type I interferon pathway genes^38^. The authors noted that M2 microglial state is responsible for mediating anti-inflammatory responses to damage caused by viral infection, but also that M2 microglial cells secrete BDNF, assisting in stimulating neural progenitor cell production and therefore promoting neurogenesis^38^. Modules Gupta_M5 and Gupta_M7 were also significantly enriched for genes from the white module, however the white module was the most highly enriched module in the Gupta_M5 gene set, while two other modules not correlated with CP status were more significantly enriched for genes from the Gupta_M7 module (Table S9).

## Discussion

Both differential expression analysis and system-level analysis of transcriptomes of a cohort of individuals with CP support perturbation of trophic signalling pathways as a common molecular abnormality. Downregulation of *NTRK2* (trkB) and *FGFR1* implicate both BDNF and FGF signalling in CP, trophic factors which play broad roles in development. FGF signalling is involved in angiogenesis, cell migration, neural outgrowth and is required for processes including normal mesoderm patterning, correct axial organisation during embryonic development and normal skeletogenesis (reviewed in ref.^39^). BDNF signalling plays roles in neuronal survival, morphogenesis and plasticity, and immune function (reviewed in refs.^40,41^). Recent evidence also suggests a role for neurotrophin signalling, including BDNF signalling, in determining pregnancy outcome, likely partly due to the role of neurotrophin signalling in placentation, with perturbed signalling observed in complicated pregnancies such as those with IUGR, pre-eclampsia and pre-term delivery (reviewed in ref.^42^). A large proportion of cases in our CP cohort were born following reported pregnancy complications (Table S1), for example, 23.6% of cases had IUGR (43 of 178 cases where data were available, defined as weight <10^th^ percentile for gestational age) and 49.7% of cases were pre-term deliveries (89 of 179 cases where data were available). Additionally, trophic signalling plays an important role in neuroprotection, with the role of BDNF and FGF signalling in recovery following hypoxic ischaemic brain injury extensively studied (reviewed in refs.^43,44^).

Several studies support a potential role for BDNF and FGF signalling in mediating damage in CP. Low level of thyroid hormone is a strong independent risk factor for white matter injury, a major cause of CP in pre-term infants. It has been shown that thyroxin treatment in post-natal rats protects against white matter injury following hypoxic ischaemic injury via upregulation of brain-derived neurotrophic factor-TrkB signalling in the immature brain^45^, therefore supporting an important role for BDNF signalling in mediating the degree of neuronal damage following developmental stresses. In addition, stress, including hypoxia, viral infection and other environmental factors, has been shown to induce the unfolded protein response/ER stress, leading to a subsequent decrease in FGFR1 expression in heart progenitor cells, which in turn has been shown to be a major contributor to congenital heart defects (CHDs)^46^. This finding is of particular interest since, similar to CP, only around 20% of CHD have a known genetic cause, with the remaining 80% suggested to result from interplay between genetics and environmental stress in utero. There is also a high rate of birth defects concurrent with CP: >40% (*n* = 185) of children with CP in one study in South Australia, compared with 4.3–5.5% for the Australian population^47^.

This study is the first large-scale analysis of transcriptional changes in CP and demonstrates the presence of convergent molecular abnormalities in clinically diverse CP, providing a basis for prioritising genes for further investigation in future genomic and functional studies. We have identified two modules of high interest for CP aetiology, one enriched for high-confidence ASD genes and the other for genes differentially expressed in CP, supporting a common molecular origin for these diverse neurodevelopmental disorders. The observed downregulation of expression of components of trophic signalling pathways and upregulation of inflammatory markers in CP may indicate increased susceptibility to neuronal damage resulting from environmental insults in utero and in early post-natal life. The role of tertiary damage following an early sensitising event, perhaps of maternal origin, including persistent inflammation and epigenetic changes, has been proposed previously as underlying a proportion of CP cases^48^ and has been suggested as a mechanism in ASD^49^. An alternative explanation for our observations is that the downregulation of trophic signalling pathways may result from chronic stress in utero or in early post-natal life. Identifying the environmental and genetic triggers of this molecular abnormality will be an important step in understanding the aetiology of CP and other neurodevelopmental disorders and predicting those at greater risk.

## Electronic supplementary material

Supplementary Document

Supplementary Table
