# Supplementary material for: Analysis of 182 cerebral palsy transcriptomes points to dysregulation of trophic signalling pathways and overlap with autism
Source: Transl Psychiatry. 2018 Apr 23;8:88. doi: 10.1038/s41398-018-0136-4 (PMC5911435; doi:10.1038/s41398-018-0136-4)
Supplement: Supplementary file 1 — Supplementary Document [file 41398_2018_136_MOESM1_ESM.pdf]

Supplementary data

Analysis of 182 cerebral palsy transcriptomes points to dysregulation of trophic signalling pathways and overlap with autism

Clare L. van Eyk,[1,2] Mark A. Corbett,[1,2] Alison Gardner,[1,2] Bregje W. van Bon,[4] Jessica L. Broadbent,[1] Kelly Harper,[1] Alastair H. MacLennan,[1] Jozef Gecz*[1,2,3]

[1] Robinson Research Institute, Faculty of Health and Medical Sciences, University of Adelaide, Adelaide, S.A., Australia

[2] Adelaide Medical School, Faculty of Health and Medical Sciences, University of Adelaide, Adelaide, S.A., Australia

[3] South Australian Health and Medical Research Institute, Adelaide, S.A., Australia

[4] Department of Human Genetics, Radboud University Medical Center, Nijmegen, the Netherlands

*Correspondence: Professor Jozef Gecz, Adelaide Medical School, Faculty of Health and Medical Sciences, University of Adelaide, Level 8, Adelaide Health and Medical Sciences Building, Corner North Terrace and George Street., Adelaide, S.A., Australia, 5005. Phone: +61 08 8313 2453, Email: jozef.gecz@adelaide.edu.au

Cell line	Proband ID	Sample type	Age at collection	Sex	RIN
4272	P001	Case	6	F	9.8
4273	P002	Case	5	F	9.7
4274	P003	Case	6	F	9.6
4275	P004	Case	5	F	9.4
4276	P005	Case	10	F	9.6
4292	P007	Case	16	F	9.6
4293	P008	Case	13	M	9.5
4305	P010	Case	8	M	9.8
4306	P011	Case	3	F	9.7
4307	P012	Case	3	F	9.6
4308	P013	Case	5	F	9.6
4309	P014	Case	7	M	9.6
4310	P015	Case	10	M	9.3
4311	P016	Case	15	M	9.2
4344	P009	Case	8	M	9.4
4345	P017	Case	6	M	9.3
4346	P018	Case	8	F	9.2
4347	P019	Case	12	M	9.4
4348	P020	Case	16	F	9.3
4349	P021	Case	4	M	9.3
4350	P022	Case	2	M	9.5
4437	P023	Case	5	F	9.2
4438	P024	Case	15	F	9.3
4569	P026	Case	15	F	9.2
4570	P027	Case	14	M	9.1
4577	P028	Case	16	F	9.3
4590	P029	Case	11	M	9.4
4591	P030	Case	18	M	9.6
4630	P031	Case	2	M	9.4
4631	P032	Case	4	M	9.5
4632	P033	Case	6	M	9.4
4633	P034	Case	10	M	9.6
4646	P035	Case	12	F	9.3
4648	P036	Case	8	F	9.5
4651	P037	Case	23	F	9.4
4681	P038	Case	32	M	9.4
4683	P025	Case	6	M	9.3
4684	P039	Case	8	M	9.3
4726	P040	Case	13	M	9.3
4727	P041	Case	20	F	9.3
4740	P042	Case	24	F	9.1
4744	P043	Case	12	F	9.1

4745	P044	Case	2	F	9
4746	P045	Case	6	F	9.2
4747	P046	Case	2	F	9.2
4748	P047	Case	10	M	9.3
4793	P049	Case	8	M	9.3
4895	P050	Case	4	M	9.4
4979	P051	Case	10	M	9.5
4984	P052	Case	15	F	9.5
5113	P053	Case	9	M	9.6
5114	P054	Case	6	M	9.6
5115	P055	Case	6	F	9.5
5116	P056	Case	10	M	9.5
5117	P057	Case	10	M	9.4
5118	P058	Case	3	M	9.5
5158	P059	Case	15	M	9.6
5239	P060	Case	3	F	9.6
5240	P061	Case	3	M	9.5
5241	P062	Case	5	F	9.4
5242	P063	Case	6	F	9.6
5243	P064	Case	6	M	9.5
5296	P065	Case	12	F	9.5
5382	P066	Case	20	M	9.5
5461	P067	Case	2	F	9.4
5494	P068	Case	6	M	9.5
5521	P069	Case	5	F	9.5
5522	P070	Case	5	M	9.4
5523	P071	Case	9	M	9.4
5524	P072	Case	10	M	9.3
5546	P073	Case	13	M	9.4
5577	P074	Case	8	F	9.3
6085	P075	Case	3	F	9.3
6086	P076	Case	6	F	8.9
6087	P077	Case	8	F	9
6123	P078	Case	2	M	9
6124	P079	Case	4	M	9.2
6125	P080	Case	18	M	9.1
6138	P081	Case	2	F	9.3
6139	P082	Case	3	F	9.4
6140	P083	Case	4	M	9.1
6141	P084	Case	11	F	9
6293	P096	Case	1	M	9.4
6294	P097	Case	2	M	9.5
6295	P098	Case	2	F	9.5
6296	P099	Case	9	M	9.4

6477	P101	Case	13	M	9.6
6478	P102	Case	17	M	9.4
6479	P103	Case	18	M	9.4
6480	P100	Case	6	F	9.5
6493	P104	Case	2	M	9.4
6494	P105	Case	2	F	9
6495	P106	Case	4	M	9.8
6496	P107	Case	4	F	9.6
6497	P108	Case	11	M	9.7
6652	P401	Case	6	F	9.7
6653	P402	Case	6	F	9.6
6654	P403	Case	7	M	9.6
6655	P404	Case	7	F	9.7
6656	P405	Case	8	M	9.7
6673	P406	Case	15	F	9.6
6674	P407	Case	4	M	9.6
6675	P408	Case	5	M	9.6
6676	P409	Case	7	M	9.5
6677	P410	Case	8	F	9.7
6680	P411	Case	13	F	9.7
6682	P109	Case	9	M	9.7
6683	P110	Case	3	M	9.6
6684	P111	Case	2	M	9.7
6685	P412	Case	18	M	9.7
6700	P413	Case	3	M	9.5
6701	P414	Case	10	M	9.6
6702	P415	Case	12	F	9.7
6703	P416	Case	12	M	9.7
6717	P417	Case	4	M	8.9
6718	P418	Case	6	F	9.3
6719	P419	Case	9	F	8.9
6720	P420	Case	10	M	9.5
6749	P422	Case	6	F	9.2
6829	P112	Case	9	F	9.2
6868	P423	Case	3	F	9.4
6869	P424	Case	4	M	9.4
6870	P425	Case	11	M	9.3
6927	P426	Case	4	F	9.4
6928	P427	Case	6	M	9.3
6929	P428	Case	9	M	9.4
6977	P429	Case	8	M	9.3
6978	P430	Case	12	F	9.3
6988	P431	Case	12	F	9.7
7016	P432	Case	8	F	9.6

7017	P433	Case	5	F	9.6
7018	P434	Case	9	F	9.6
7028	P435	Case	11	M	9.6
7055	P436	Case	4	F	9.5
7056	P437	Case	9	M	9.3
7057	P438	Case	12	M	9.6
7058	P439	Case	14	M	9.5
7075	P113	Case	7	M	9.5
7132	P440	Case	6	M	9.5
7133	P441	Case	8	M	9.5
7134	P442	Case	8	M	9.4
7135	P443	Case	9	F	9.3
7198	P444	Case	6	F	9.2
7199	P445	Case	9	M	9.4
7200	P446	Case	4	F	9.3
7209	P447	Case	11	M	9.4
7210	P448	Case	12	M	9.4
7211	P449	Case	16	F	9.3
7268	P450	Case	5	M	9.5
7269	P451	Case	8	F	9.2
7270	P452	Case	13	M	9.4
7315	P114	Case	2	F	9.3
7322	P453	Case	3	F	9.7
7323	P454	Case	3	M	9.8
7370	P455	Case	5	M	9.8
7371	P456	Case	4	M	9.8
7372	P457	Case	14	M	9.8
7385	P458	Case	5	F	9.7
7386	P459	Case	4	F	9.7
7387	P460	Case	14	M	9.7
7388	P461	Case	8	M	9.7
7420	P462	Case	10	F	9.6
7421	P463	Case	11	F	9.6
7492	P421	Case	13	F	9.8
7495	P464	Case	6	M	9.7
7501	P465	Case	4	F	9.6
7502	P466	Case	8	M	9.7
7509	P115	Case	3	M	9.6
7510	P116	Case	4	F	9.6
7511	P117	Case	4	M	9.5
7526	P467	Case	6	F	9.1
7527	P468	Case	12	F	9.6
7528	P469	Case	11	F	9.6
7646	P470	Case	3	M	9.6

7647	P471	Case	4	F	9.5
7648	P472	Case	13	M	9.6
7772	P474	Case	3	M	9.6
7773	P475	Case	5	M	9.6
7775	P118	Case	7	M	9.4
7939	P476	Case	6	F	9.5
7957	P119	Case	12	F	9.5
7997	P477	Case	7	F	9.5
TW	94595	Control	42	F	9.6
JD	83921	Control	49	M	9.7
KQ	23397	Control	47	M	9.5
VB	88981	Control	59	M	9.7
AB	22121	Control	41	M	9.6
MH	17097	Control	44	F	9.6
LW	88804	Control	32	F	9.5
ET	80795	Control	72	M	9.5
TB	90653	Control	28	F	9.6
JuL	90185	Control	69	F	9.6
RA	91119	Control	40	F	9.5
JoL	90185	Control	73	M	9.6
MD	86598	Control	65	F	9.8
JP	83606	Control	77	F	9.8
AH	87509	Control	4	M	10
PK	85983	Control	39	M	9.6
JG	84986	Control	63	F	9.8
RS	23398	Control	27	M	9.5
BH	84116	Control	45	M	9.5
KW	95824	Control	37	F	9.6

Table S2: RNA samples sequenced in this study. RIN, RNA integrity number; F, female; M, male.

Sample ID	Population	Sex
HG00096	GBR	M
HG00099	GBR	F
HG00102	GBR	F
HG00105	GBR	M
HG00108	GBR	M
HG00110	GBR	F
HG00111	GBR	F
HG00114	GBR	M
HG00119	GBR	M
HG00120	GBR	F
HG00121	GBR	F
HG00122	GBR	F
HG00123	GBR	F
HG00124	GBR	F
HG00125	GBR	F
HG00126	GBR	M
HG00127	GBR	F
HG00128	GBR	F
HG00129	GBR	M
HG00130	GBR	F
HG00131	GBR	M
HG00133	GBR	F
HG00135	GBR	F
HG00136	GBR	M
HG00137	GBR	F
HG00138	GBR	M
HG00142	GBR	M
HG00148	GBR	M
HG00150	GBR	F
HG00151	GBR	M
HG00155	GBR	M
HG00156	GBR	M
HG00160	GBR	M
HG00171	FIN	F
HG00173	FIN	F
HG00176	FIN	F
HG00179	FIN	F
HG00181	FIN	M
HG00186	FIN	M
HG00189	FIN	M
HG00232	GBR	F
HG00235	GBR	F
HG00242	GBR	M
HG00243	GBR	M
HG00247	GBR	F
HG00249	GBR	F
HG00250	GBR	F

Sample ID	Population	Sex
HG00252	GBR	M
HG00261	GBR	F
HG00262	GBR	F
HG00263	GBR	F
HG00274	FIN	F
HG00276	FIN	F
HG00278	FIN	M
HG00319	FIN	F
HG00323	FIN	F
HG00329	FIN	M
HG00350	FIN	F
HG00351	FIN	M
HG01790	GBR	F
NA06984	CEU	M
NA06989	CEU	F
NA07048	CEU	M
NA10851	CEU	M
NA11829	CEU	M
NA11830	CEU	F
NA11992	CEU	M
NA12342	CEU	M
NA12348	CEU	F
NA12399	CEU	M
NA12400	CEU	F
NA12546	CEU	M
NA12749	CEU	F
NA12760	CEU	M
NA12762	CEU	M
NA12777	CEU	M
NA12812	CEU	M
NA12827	CEU	M
NA12830	CEU	F
NA12843	CEU	F
NA12874	CEU	M
NA12890	CEU	F
NA18487	YRI	M
NA18517	YRI	F
NA18861	YRI	M
NA19119	YRI	M
NA20502	TSI	F
NA20504	TSI	F
NA20506	TSI	F
NA20510	TSI	M
NA20520	TSI	M
NA20542	TSI	F
NA20787	TSI	M
NA20805	TSI	M

Table S3: Samples from the gEUVADIS data set used in this study. Sample IDs refer to the individual as listed in the 1000 genomes project database http://www.1000genomes.org/. F, female; M, male.

Gene Symbol	Log Fold Change	Average Log Expression	Adjusted p value
MIR650	3.38	5.40	7.18E-12
KLHL14	2.65	3.13	7.95E-31
AEBP1	2.07	3.51	2.66E-26
RASGRP2	1.97	3.77	4.19E-50
RBMS1	1.93	3.07	1.93E-34
PXDN	1.91	4.06	1.01E-16
PLD4	1.90	3.80	7.90E-34
CD24	1.89	4.85	1.30E-28
FAM46C	1.75	6.06	1.49E-27
ZFY	1.72	3.59	9.75E-09
IGJ	1.71	10.63	2.43E-19
ARL4C	1.69	4.77	1.40E-24
LOC96610	1.68	7.10	1.83E-20
MTSS1	1.65	3.42	2.80E-27
RSAD2	1.58	4.83	1.67E-31
DDX60L	1.58	5.11	3.44E-30
IGLL5	1.57	10.11	3.30E-14
DDX3Y	1.54	6.94	3.92E-11
VPREB3	1.53	3.02	5.87E-14
ZAP70	1.53	2.48	2.42E-14
KLF2	1.53	4.42	1.99E-32
USP9Y	1.51	5.34	1.92E-12
ZBP1	1.50	5.04	4.59E-37
PDE3B	1.40	3.18	5.40E-11
RALGPS2	1.37	6.19	9.82E-35
CD180	1.36	5.99	1.68E-32
UTY	1.36	5.00	3.63E-11
CD28	1.35	4.63	2.49E-12
SEL1L3	1.31	7.81	4.56E-26
TXNIP	1.28	7.31	3.82E-21
CMPK2	1.27	5.59	5.10E-28
TXLNG2P	1.23	5.50	1.94E-10
CECR1	1.23	6.85	9.34E-33
IFIT1	1.23	6.78	2.69E-21
JHDM1D	1.22	3.39	1.65E-26
NEFH	1.22	3.57	1.53E-17
C17orf28	1.21	2.99	5.05E-19
FOXP1	1.21	3.11	1.42E-11
CD38	1.20	6.05	5.41E-20
LPAR6	1.20	5.31	2.28E-14
LGALS3BP	1.19	5.04	4.08E-14
FER1L4	1.16	3.30	3.80E-25
C8orf80	1.16	4.43	2.27E-21
CD96	1.16	4.13	4.86E-08

Gene Symbol	Log Fold Change	Average Log Expression	Adjusted p value
HIP1R	1.14	5.26	3.10E-27
PLAC8	1.11	7.74	8.93E-19
ARRDC3	1.09	5.14	1.81E-17
KCNA3	1.09	3.84	3.93E-20
CPM	1.09	3.87	2.37E-19
FCRL4	1.06	2.80	1.03E-07
LTB	1.05	5.52	9.33E-19
MPEG1	1.05	5.69	6.54E-20
CPNE5	1.04	6.11	9.25E-23
FCRL5	1.04	7.93	1.33E-22
IFI27	1.04	2.84	3.45E-06
BCL11A	1.03	4.76	4.65E-23
CD72	1.02	4.51	6.52E-27
ISG15	1.02	8.38	1.82E-22
BIK	1.01	3.22	1.14E-13
NEIL1	1.01	3.86	5.21E-20
CCR1	1.00	4.16	5.49E-12
IGFBP4	-1.00	3.33	3.65E-07
CD80	-1.01	5.93	2.66E-28
DAPK1	-1.01	6.09	3.47E-17
PVRL1	-1.02	4.26	3.60E-15
RPS4Y1	-1.02	6.56	0.000271
ADCY6	-1.02	3.62	1.59E-14
SPATS2L	-1.03	5.13	3.87E-14
BHLHE40	-1.03	7.23	5.21E-14
ACTA2	-1.04	4.50	1.65E-15
FSCN1	-1.04	8.09	2.44E-17
ABCB4	-1.04	3.33	3.98E-24
CACNB4	-1.04	3.85	2.60E-06
CLIC2	-1.04	3.38	1.18E-11
DENND3	-1.05	6.26	3.05E-25
PCGF2	-1.06	3.10	3.18E-11
PPFIBP1	-1.06	5.13	3.19E-21
KIAA1324L	-1.06	3.29	1.07E-12
CDKN1A	-1.06	8.11	1.15E-38
PTAFR	-1.06	5.40	5.20E-08
CTH	-1.07	4.24	2.37E-10
NID1	-1.08	5.06	4.34E-11
BAZ2B	-1.08	4.74	2.00E-32
ARHGAP10	-1.08	4.95	2.64E-34
EBI3	-1.09	6.76	4.27E-17
CPNE8	-1.09	4.55	4.96E-32
ANK1	-1.10	4.08	4.62E-11
INPP5F	-1.10	2.81	1.76E-11

PLAG1	-1.11	2.81	1.32E-17
LAG3	-1.12	3.86	2.26E-09
RNF207	-1.12	3.32	7.38E-27
CNTNAP1	-1.12	6.01	7.38E-27
AMOTL1	-1.13	3.33	1.18E-08
CCDC74A	-1.13	3.08	4.65E-05
CDC42BPA	-1.14	4.65	4.54E-06
GPR15	-1.15	6.56	1.43E-39
RGS2	-1.15	3.07	7.81E-08
PAPLN	-1.16	4.00	1.38E-21
IL2RA	-1.16	3.24	1.08E-08
TMEM132A	-1.16	2.81	2.25E-16
IL4I1	-1.17	6.48	4.25E-08
TNFRSF8	-1.17	6.57	5.37E-33
CCL22	-1.17	8.96	2.09E-10
TEX9	-1.18	4.05	8.95E-20
MB21D2	-1.18	3.86	6.06E-07
IL12RB2	-1.19	2.94	6.14E-10
TMTC2	-1.19	2.93	9.05E-25
TNIK	-1.20	6.62	4.49E-18
TNFAIP2	-1.21	5.51	4.50E-18
TBX15	-1.24	3.13	1.99E-06
SLC12A8	-1.24	5.63	1.51E-16
PCNXL2	-1.24	2.91	2.27E-26
PRKCH	-1.25	3.89	2.18E-07
PRRT3	-1.25	3.96	1.80E-38
AUTS2	-1.26	4.38	1.61E-12
EIF1AY	-1.26	4.63	0.005482
LRRK2	-1.27	5.34	1.08E-10
RASSF4	-1.29	4.81	1.33E-29
NACC2	-1.29	3.34	2.07E-18
PARM1	-1.30	5.00	1.61E-10
GIMAP6	-1.32	4.95	2.79E-18
CBS	-1.33	2.70	6.56E-12
PHLDA3	-1.33	4.07	1.40E-19
CSF1	-1.39	4.82	1.72E-25
GBP4	-1.40	4.80	2.39E-12
RAB38	-1.41	3.31	6.11E-15
RGS16	-1.41	5.62	7.48E-43
ITGB8	-1.41	4.21	3.01E-17
NCALD	-1.41	4.64	1.08E-20
SOCS3	-1.43	3.44	7.43E-11
ATF5	-1.46	6.94	4.32E-12
HDGFRP3	-1.47	4.35	1.02E-20
DNAH17	-1.50	3.43	2.03E-14

EDA2R	-1.50	4.21	3.06E-48
KIAA2022	-1.51	2.83	2.23E-20
STARD13	-1.52	4.44	1.50E-35
LIPH	-1.54	3.21	5.89E-24
HAAO	-1.55	3.74	2.04E-42
SV2A	-1.59	2.86	4.34E-25
NTRK2	-1.59	3.43	6.88E-26
ROBO1	-1.64	4.14	2.95E-13
GPR55	-1.65	4.61	2.59E-25
FAM169A	-1.69	2.92	3.18E-21
TOX	-1.69	3.20	7.59E-15
TNFSF4	-1.72	3.89	5.21E-20
HCK	-1.72	3.68	2.58E-09
ANO10	-1.72	3.19	7.43E-30
BAIAP2L1	-1.73	3.55	3.53E-14
ZNF697	-1.76	2.78	1.14E-33
TMOD1	-1.81	3.67	1.71E-22
MOXD1	-1.83	3.39	6.92E-15
UNC13B	-1.84	2.90	1.05E-15
IL18R1	-1.84	4.07	9.08E-23
TSPAN12	-1.86	3.51	2.53E-31
RIMS3	-1.88	2.66	9.05E-25
ADAM22	-1.90	3.96	2.62E-60
HECW2	-1.96	3.33	2.85E-20
PARD3	-1.96	4.13	4.93E-21
UCHL1	-1.96	4.23	2.49E-15
ACTN1	-1.99	4.23	3.78E-21
GSTM1	-1.99	2.85	1.10E-07
LOXL3	-2.00	3.29	9.19E-35
SMAD1	-2.03	3.41	1.71E-27
ARHGAP6	-2.18	3.02	2.25E-25
CACNA1E	-2.25	4.08	5.64E-28
FGFR1	-2.30	4.05	2.92E-29
ARHGEF17	-2.32	3.54	9.23E-33
CYP1B1	-2.37	4.50	5.72E-29
APBB2	-2.46	2.85	2.10E-25
TNFRSF19	-2.49	3.53	3.94E-17
CNR1	-2.59	3.17	3.70E-27
LOC728175	-2.61	3.52	5.16E-30
LAMP5	-2.65	4.15	8.22E-14
EVC	-2.75	3.21	5.71E-29
DST	-3.04	6.75	6.90E-54
FBN1	-3.07	4.14	4.81E-63
KIF21A	-3.12	4.11	4.03E-47

Table S4: Genes differentially expressed between Cerebral Palsy cases and controls. Differential expression analysis performed on batch normalised data (ComBat) using the EdgeR package from Bioconductor.

Gene Symbol	Log Fold Change	Average Log Expression	Adjusted P value
MIR650	3.38	5.40	7.18E-12
KLHL14	2.65	3.13	7.95E-31
AEBP1	2.07	3.51	2.66E-26
RASGRP2	1.97	3.77	4.19E-50
RBMS1	1.93	3.07	1.93E-34
PLD4	1.90	3.80	7.90E-34
CD24	1.89	4.85	1.30E-28
FAM46C	1.75	6.06	1.49E-27
ZFY	1.72	3.59	9.75E-09
IGJ	1.71	10.63	2.43E-19
ARL4C	1.69	4.77	1.40E-24
LOC96610	1.68	7.10	1.83E-20
MTSS1	1.65	3.42	2.80E-27
RSAD2	1.58	4.83	1.67E-31
DDX60L	1.58	5.11	3.44E-30
IGLL5	1.57	10.11	3.30E-14
KLF2	1.53	4.42	1.99E-32
ZBP1	1.50	5.04	4.59E-37
PDE3B	1.39	3.18	5.40E-11
RALGPS2	1.37	6.19	9.82E-35
CD180	1.36	5.99	1.68E-32
CD28	1.35	4.63	2.49E-12
SEL1L3	1.31	7.81	4.56E-26
TXNIP	1.28	7.31	3.82E-21
CMPK2	1.27	5.59	5.10E-28
CECR1	1.23	6.85	9.34E-33
IFIT1	1.23	6.78	2.69E-21
JHDM1D	1.22	3.39	1.65E-26
C17orf28	1.21	2.99	5.05E-19
FOXP1	1.21	3.11	1.42E-11
CD38	1.20	6.05	5.41E-20
LPAR6	1.20	5.31	2.28E-14
LGALS3BP	1.19	5.04	4.08E-14
FER1L4	1.16	3.30	3.80E-25
C8orf80	1.16	4.43	2.27E-21
HIP1R	1.14	5.26	3.10E-27
PLAC8	1.11	7.74	8.93E-19
ARRDC3	1.09	5.14	1.81E-17
KCNA3	1.09	3.84	3.93E-20
CPM	1.09	3.87	2.37E-19
FCRL4	1.06	2.80	1.03E-07
LTB	1.05	5.52	9.33E-19
MPEG1	1.05	5.69	6.54E-20

Gene Symbol	Log Fold Change	Average Log Expression	Adjusted P value
CPNE5	1.04	6.11	9.25E-23
FCRL5	1.04	7.93	1.33E-22
IFI27	1.04	2.84	3.45E-06
BCL11A	1.03	4.76	4.65E-23
ISG15	1.02	8.38	1.82E-22
BIK	1.01	3.22	1.14E-13
PECAM1	1.00	3.19	4.01E-23
IGFBP4	-1.00	3.33	3.65E-07
CD80	-1.01	5.93	2.66E-28
DAPK1	-1.01	6.09	3.47E-17
PVRL1	-1.02	4.26	3.60E-15
ADCY6	-1.02	3.62	1.59E-14
SPATS2L	-1.03	5.13	3.87E-14
BHLHE40	-1.03	7.23	5.21E-14
ACTA2	-1.04	4.50	1.65E-15
FSCN1	-1.04	8.09	2.44E-17
ABCB4	-1.04	3.33	3.98E-24
CACNB4	-1.04	3.85	2.60E-06
CLIC2	-1.04	3.38	1.18E-11
DENND3	-1.05	6.26	3.05E-25
PCGF2	-1.06	3.10	3.18E-11
PPFIBP1	-1.06	5.13	3.19E-21
CDKN1A	-1.06	8.11	1.15E-38
PTAFR	-1.06	5.40	5.20E-08
CTH	-1.07	4.24	2.37E-10
NID1	-1.08	5.06	4.34E-11
BAZ2B	-1.08	4.74	2.00E-32
ARHGAP10	-1.08	4.95	2.64E-34
EBI3	-1.09	6.76	4.27E-17
CPNE8	-1.09	4.55	4.96E-32
ANK1	-1.10	4.08	4.62E-11
INPP5F	-1.10	2.81	1.76E-11
PLAG1	-1.11	2.81	1.32E-17
LAG3	-1.12	3.86	2.26E-09
RNF207	-1.12	3.32	7.38E-27
CNTNAP1	-1.12	6.01	7.38E-27
AMOTL1	-1.13	3.33	1.18E-08
CDC42BPA	-1.14	4.65	4.54E-06
GPR15	-1.15	6.56	1.43E-39
PAPLN	-1.16	4.00	1.38E-21
IL2RA	-1.16	3.24	1.08E-08
TMEM132A	-1.16	8.06	4.46E-08
IL4I1	-1.17	6.48	4.25E-08

TNFRSF8	-1.17	6.57	5.37E-33
CCL22	-1.17	8.96	2.09E-10
TEX9	-1.18	4.05	8.95E-20
TNIK	-1.20	6.62	4.49E-18
TNFAIP2	-1.21	5.51	4.50E-18
TBX15	-1.24	3.13	1.99E-06
SLC12A8	-1.24	5.63	1.51E-16
PCNXL2	-1.24	2.91	2.27E-26
PRKCH	-1.25	3.89	2.18E-07
PRRT3	-1.25	3.96	1.80E-38
AUTS2	-1.26	4.38	1.61E-12
LRRK2	-1.27	5.34	1.08E-10
PARM1	-1.30	5.00	1.61E-10
GIMAP6	-1.32	4.95	2.79E-18
CBS	-1.33	2.70	6.56E-12
PHLDA3	-1.33	4.07	1.40E-19
CSF1	-1.39	4.82	1.72E-25
RAB38	-1.41	3.31	6.11E-15
RGS16	-1.41	5.62	7.48E-43
ITGB8	-1.41	4.21	3.01E-17
NCALD	-1.41	4.64	1.08E-20
SOCS3	-1.43	3.44	7.43E-11
DNAH17	-1.50	3.43	2.03E-14
EDA2R	-1.50	4.21	3.06E-48
KIAA2022	-1.51	2.83	2.23E-20
STARD13	-1.52	4.44	1.50E-35
LIPH	-1.54	3.21	5.89E-24
HAAO	-1.55	3.74	2.04E-42
SV2A	-1.59	2.86	4.34E-25
NTRK2	-1.59	3.43	6.88E-26
ROBO1	-1.64	4.14	2.95E-13
GPR55	-1.65	4.61	2.59E-25
TOX	-1.69	3.20	7.59E-15

TNFSF4	-1.72	3.89	5.21E-20
HCK	-1.72	3.68	2.58E-09
ANO10	-1.72	3.19	7.43E-30
BAIAP2L1	-1.73	3.55	3.53E-14
ZNF697	-1.76	2.78	1.14E-33
TMOD1	-1.81	3.67	1.71E-22
MOXD1	-1.83	3.39	6.92E-15
UNC13B	-1.84	2.90	1.05E-15
IL18R1	-1.84	4.07	9.08E-23
RIMS3	-1.88	2.66	9.05E-25
ADAM22	-1.90	3.96	2.62E-60
HECW2	-1.96	3.33	2.85E-20
PARD3	-1.96	4.13	4.93E-21
ACTN1	-1.99	4.23	3.78E-21
GSTM1	-1.99	2.85	1.10E-07
LOXL3	-2.00	3.29	9.19E-35
SMAD1	-2.03	3.41	1.71E-27
ARHGAP6	-2.18	3.02	2.25E-25
CACNA1E	-2.25	4.08	5.64E-28
FGFR1	-2.30	4.05	2.92E-29
ARHGEF17	-2.32	3.54	9.23E-33
CYP1B1	-2.37	4.50	5.72E-29
APBB2	-2.46	2.85	2.10E-25
TNFRSF19	-2.49	3.53	3.94E-17
CNR1	-2.59	3.17	3.70E-27
LOC728175	-2.61	3.52	5.16E-30
LAMP5	-2.65	4.15	8.22E-14
EVC	-2.75	3.21	5.71E-29
DST	-3.04	6.75	6.90E-54
FBN1	-3.07	4.14	4.81E-63
KIF21A	-3.12	4.11	4.03E-47

Table S5: Significant differentially expressed genes (Fold change ≥2, ≤-2) common to both cerebral palsy cases compared to in-house and gEUVADIS control data sets and cerebral palsy cases compared to in-house control samples regressed for age. Differential expression analysis was performed using the EdgeR package from Bioconductor.

**Cell line ID**	**Age at collection**	**Sex**
4611	19	F
4669	22	M
4673	26	M
4701	26	F
4706	25	F
4750	21	M
4768	23	F
4910	21	M
5004	20	M
6931	25	F

Table S6: Control samples used for validation of gene expression changes. Samples are from the 'Aussie Normals' collection (https://www.neura.edu.au/scientific-facility/gra/biorepository-catalog/)

Sample ID	***Total number of outliers with cut-off of ±4SDs***
P058	*4*
P409	*9*
P425	*25*
P459	*9*
P463	*19*
P467	*17*
P469	*6*
P470	*5*
P429	*1*

Table S7: Total number of outliers with cut-off of ±4SDs in samples where additional genetic variants were identified by correlation of gene expression changes with genetic data.

**Modules from the CP unsigned network**	**Voineagu *et al.* combined network module**	**Enrichment p-value (corrected p-value)**	**Voineagu *et al.* annotation and cell type marker**
black	M9_lightgreen	$1.90x10^{-23}$ ($3.42x10^{-22}$)	Ribosome
brown	M16_tan	$1.30x10^{-5}$ ($2.33x10^{-4}$)	Astrocytes, activated microglia
cyan	M17_turquoise	$4.4x10^{-2}$ (ns)	Neurons
grey60	ns	ns	Ns
lightyellow	M16_tan	$2.34x10^{-4}$ ($4.22x10^{-3}$)	Astrocytes, activated microglia
magenta	M3_brown	$1.97x10^{-3}$ ($3.54x10^{-2}$)	Ns
midnightblue	M2_blue	$4.34x10^{-34}$ ($7.82x10^{-33}$)	neurons
pink	ns	ns	Ns
purple	M6_greenyellow	$7.27x10^{-8}$ ($1.31x10^{-6}$)	Ns
red	M17_turquoise	$2.91x10^{-3}$ (ns)	neurons
turquoise	M18_yellow	$9.20x10^{-14}$ ($1.66x10^{-12}$)	Synapse, glutamergic neurons
white	M16_tan	$1.19x10^{-2}$ (ns)	Astrocytes, activated microglia
yellow	M10_magenta, M13_purple, M16_tan	$2.50x10^{-61}$ ($4.50x10^{-60}$), $3.23x10^{-27}$ ($5.81x10^{-26}$), $3.60x10^{-18}$ ($6.48x10^{-17}$)	Astrocytes, activated microglia

Table S8: Enrichment of our network modules for genes from autism cortex network modules. The autism unsigned co-expression network was reconstructed with data from Voineagu *et al* 2011 with parameters for WGCNA as reported.

**Modules enriched for M16_tan module signal (Gupta *et al.)***	**Module from CP unsigned network**	**Corrected P-value for enrichment**	**Module eigengene significance - CP status**	**Annotation and cell type marker enrichment**
**M5**	**white**	**$8.19x10^{-8}$**	**$1.59x10^{-9}$**	**Microglia, BDNF signalling**
	magenta	$8.96x10^{-8}$	$3.88x10^{-4}$	Protein processing in ER
	brown	$9.00x10^{-3}$	ns	Microglia, Astrocytes, Interferon alpha and beta signalling
	lightyellow	$3.64x10^{-2}$	$3.31x10^{-3}$	Protein processing in ER
**M7**	turquoise	$2.91x10^{-9}$	ns	Glu neurons, Glutamergic synaptic function
	black	$2.27x10\text{-}^{8}$	ns	Microglia, Eukaryotic elongation reactome
	white	$4.64x10^{-5}$	$1.59x10^{-9}$	Microglia, BDNF signalling
	brown	0.001802	ns	Microglia, Astrocytes, Interferon alpha and beta signalling

Table S9: Enrichment of our network modules for genes from cortex modules enriched with signal from the M16_tan network from the larger RNA sequencing study from Gupta *et al.* 2014. The M5 module was most enriched with genes from the white module from our network.

A

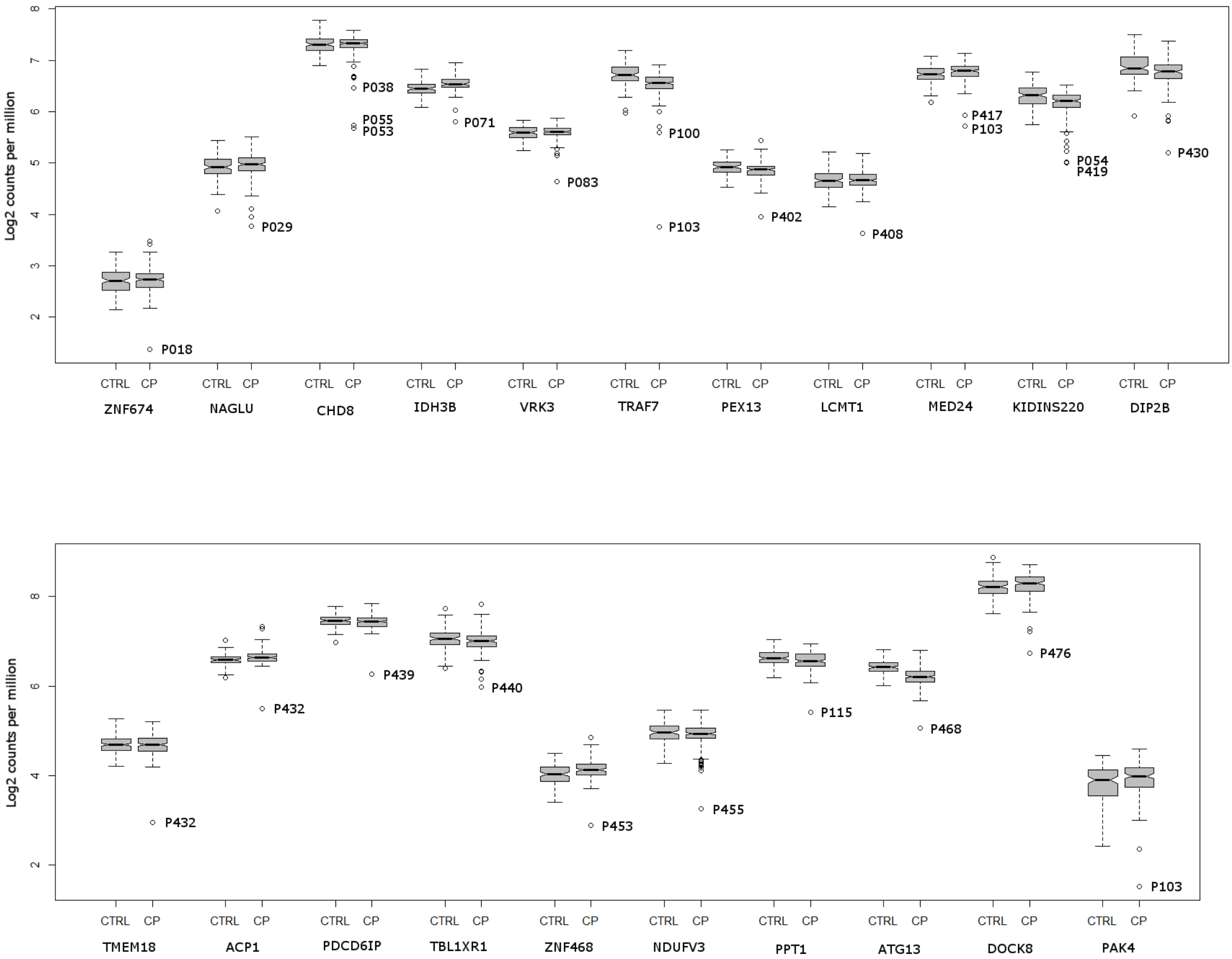

B

Gene	Case ID	Base pairs with >20x coverage (percent)	Median coverage per base pair	Mean coverage per base pair
ACP1	P432	84.68%	38	37.2
ATG13	P468	94.78%	49	53.2
CHD8	P038	88.74%	67	33.9
CHD8	P053	88.91%	85	92.8
CHD8	P055	89.03%	75	82.2
DIP2B	P430	96.02%	44	47.2
DOCK8	P476	83.92%	57	60.4
IDH3B	P078	100.00%	86	85.1
KIDINS220	P054	98.91%	87	104.5
KIDINS220	P419	98.63%	73	84.0
LCMT1	P408	94.69%	53	57.4
MED24	P103	100.00%	68	69.5
MED24	P417	100.00%	61	65.4
NAGLU	P029	78.24%	41	43.2
NDUFV3	P455	88.50%	57	54.9
PAK4	P103	82.74%	47	50.4
PDCD6IP	P439	95.73%	56	28.5
PEX13	P402	95.15%	53	52.2
PPT1	P115	100.00%	77	85.6
TBL1XR1	P440	100.00%	98	98.6
TMEM18	P432	57.94%	43	35.0
TRAF7	P100	98.08%	62	67.2
VRK3	P083	89.28%	60	62.0
ZNF468	P453	21.50%	14	14.2
ZNF674	P018	100.00%	110	166.5

Figure S1: Outliers of interest with no detected genetic variant from WES to explain gene expression. Samples had expression more than 4 standard deviations from the mean. **A)** Boxplots of log2 counts per million for genes of interest. Samples with expression more than 4 standard deviations from the mean are listed. **B)** Whole exome sequencing coverage of genes of interest in outlier samples.

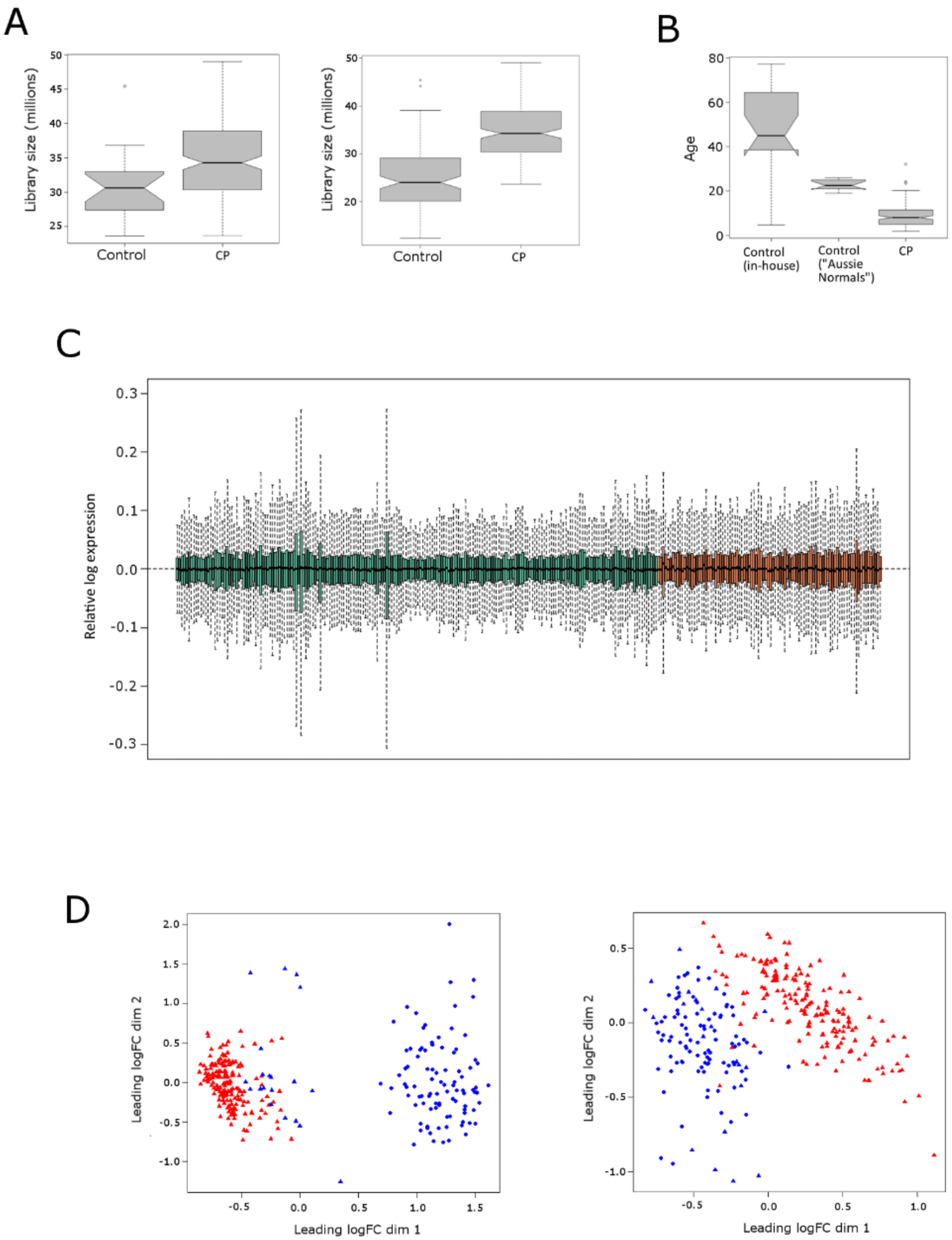
A
Library size (millions)
Control
CP
B
Age
Control (in-house)
Control ("Aussie Normals")
CP
C
Relative log expression
D
Leading logFC dim 2
Leading logFC dim 1

Figure S2: A) RNA sequencing library sizes. Left panel is samples sequenced as part of this study (all CP samples and 20 in-house controls). Right panel is all samples, including the 100 gEUVADIS data set controls. **B)** Age profile at time of sampling for samples from this study. In-house controls are the 20 control samples used in RNA sequencing, while the 'Aussie Normals' collection controls were used for quantitative RT PCR validation of select differentially expressed genes. **C)** Relative log expression of transcripts in each sample following data normalisation and batch correction using ComBat function from the SVA package in R. Green samples are those sequenced in this study, while orange samples are samples sequenced as part of the gEUVADIS data set. **D)** Multidimensional scaling plots for all samples before (left) and after (right) gender effects were blocked and batch effects removed using the ComBat function. CP cases are in red and controls are in blue, with triangles for samples sequenced in this study and circles for samples from the gEUVADIS data set.

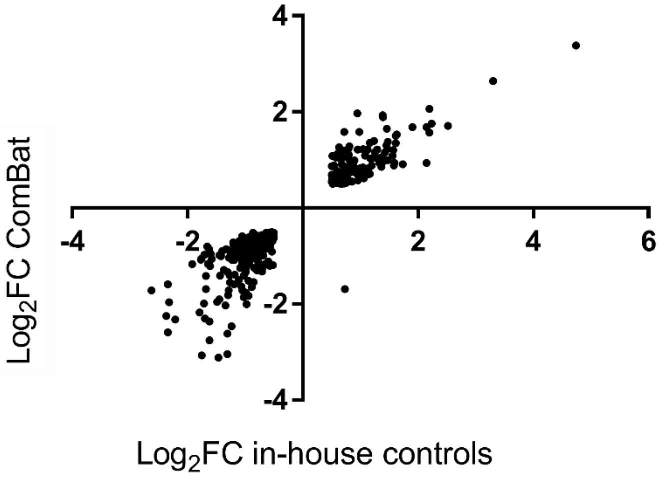

Figure S3: Concordance of Log_2FC for CP differentially expressed genes common to ComBat normalised data including samples from the gEUVADIS data set and data with in-house controls only. Only one gene of the 387 DE genes, *FAM169A*, showed discordance in direction of expression change.

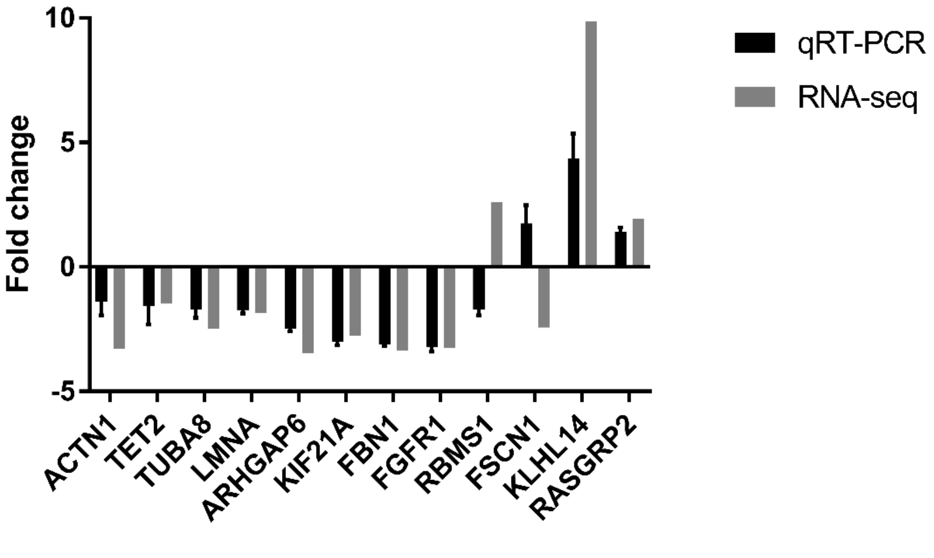

Figure S4: Quantitative Real Time PCR validation of genes differentially expressed in CP cases compare to Controls. Reverse transcription was performed on pooled RNA from 5 LCL samples, with a total of 2 control pools and 5 CP pools. Bar plots show fold change in CP cases compared to controls, with black bars showing fold change from qRT-PCR and grey bars showing fold change from RNA seq for CP cases compared to in-house controls.
