# Supplementary material for: Analysis of 182 cerebral palsy transcriptomes points to dysregulation of trophic signalling pathways and overlap with autism
Source: Transl Psychiatry. 2018 Apr 23;8:88. doi: 10.1038/s41398-018-0136-4 (PMC5911435; doi:10.1038/s41398-018-0136-4)
Supplement: Supplementary file 2 — Supplementary Table [file 41398_2018_136_MOESM2_ESM.docx]

| **Cell line** | **Proband**  **ID** | **Sex** | **Gestation (weeks)** | **CP type** | **GMFCS** | **Plurity** | **Age at blood collection** | **Clinical details for pregnancy** | **Proband clinical details and neuroimaging** | **Genetic/expression data** | **EASE GO enrichment of outliers from RNA seq** |
| --- | --- | --- | --- | --- | --- | --- | --- | --- | --- | --- | --- |
| 4272 | P001 | F | 25 | SpQ | 5 | S | 6 | IUGR | Epilepsy, bronchopulmonary dysplasia, bilateral Grade III and IV IVH |  | kinase activity, organogenesis, morphognenesis, development, neurogenesis |
| 4273 | P002 | F | 29 | Asym/SpD | 1 | Tw | 5 | Smoking, recreational drugs (marijuana) | Mild speech delay |  | regulation of transcription |
| 4274 | P003 | F | 32 | Dys/SpD | 4 | Tw | 6 | Herpes 21+ weeks | Microcephaly, convergent squint, cortical visual impairment, Epilepsy, some cognitive impairment |  | Receptor signalling protein activity, regulation of transcription |
| 4275 | P004 | F | 40 | SpH | 2 | S | 5 |  | Porencephalic cyst, 2 siblings of father with ID |  | antiviral response protein activity, motor activity, golgi apparatus, microtubule associated complex |
| 4276 | P005 | F | 32 | H |  | S | 10 | IUGR | Grade I IVH |  | cell communication, cytokine binding, response to biotic stimulus |
| 4292 | P007 | F | 32 | A/SpD | 2 | Tw | 16 | IUGR | Borderline intellectual impairment, colpocephaly | CDK17 (de novo, F/shift ins)  z-score=-2.24 | protein-nucleus import, docking |
| 4293 | P008 | M | 34 | SpT | 2 | S | 13 |  | Right orchidopexy |  | receptor activity, signal transducer activity, cell communication |
| 4305 | P010 | M | 40 | SpD | 5 | S | 8 |  | Severe ID and global DD, ASD, hydrocephalus and aqueduct stenosis, visual impairment suspected of cerebral origin with roving eyes and visual inattention. Self-injuring behaviour and sleep disturbances | MCPH1 (inherited from Father, clinical significance unclear) | kinase activity, xenobiotic metabolism |
| 4306 | P011 | F | 30 | SpD | 3 | S | 3 |  |  |  | cell cycle, kinase activity |
| 4307 | P012 | F | 29 | Asym/D | 4 | Tw | 3 | Gastric infection 21+ weeks | ASD, mild ID, No communication, verbal or non-verbal |  | cell-cell signalling, defense response, immune response |
| 4308 | P013 | F | 39 | SpH | 2 | S | 5 | Aspirin use during pregnancy, Cold/flu 1-20 weeks | Mild ID, ASD, intractable partial epilepsy, microcephaly, Left MCA and partial anterior cerebral artery territory infarct |  | regulation of transcription, signal transducer activity, TGF beta receptor signalling |
| 4309 | P014 | M | 30 | SpQ | 4 | Tw | 7 | Burst appendix 24 weeks, IUGR |  |  | RNA modification, heme biosynthesis |
| 4310 | P015 | M | 29 | DH | 2 | S | 10 | Early onset severe pre-eclampsia, IUGR | Epilepsy, cognitive ability upper limit of low-average range, developed hydrocephalus, grade 4 IVH, right sided, and grade 2 IVH left sided | PAK3 (X-linked) | GTP binding, negative regulation of cell proliferation |
| 4311 | P016 | M | 40 | SpQ | 5 | S | 15 | Fever within 1 week after birth, IUGR | Epilepsy, tonic-clonic seizures 2-3 times per day, improved by ketogenic diet, severe ID, communicates non-verbally | PRODH (CH) | amino acid transport (solute transporters) |
| 4344 | P009 | M | 34 | SpD | 2 | S | 8 | Possible Grave's disease (hyperthyroidism), Cold/flu 21+ weeks | Squint, long-sighted, very sensitive hearing, mild learning problems, Born with mild pulmonary stenosis and patent ductus arteriosis, evaluated without residual findings | MAST1 (de novo) | proteasome core complex, sodium ion transport, peptidase activity |
| 4345 | P017 | M | 39 | Dys/SpQ | 4 | S | 6 |  | Microcephaly due to cortical dysplasia, severe polymicrogyria, DD, epilepsy, double aortic arch | DYNC1H1 (de novo) | golgi apparatus, response to wounding, oxidoreductase activity |
| 4346 | P018 | F | 29 | D | 2 | S | 8 | Fever 1-20 weeks | ASD, squint | KIF1A (CH), **ZNF674** z-score= -4.00, associated with XLMR | ribonucleoprotein complex, proteasome complex, mitochondrion |
| 4347 | P019 | M | 37 | D | 2 | S | 12 | Malnourishment, Cold/flu 1-20 weeks | Mild asymmetry of the hypothalamus, findings support the presence of a neuroepithelial cyst | TENM1 (X-linked), ILRAPL1 (X-linked)  ATP1B4 (X-linked) | Arp2/3 protein complex, actin cytoskeleton, frizzled signaling pathway, circulation |
| 4348 | P020 | F | 40 | H | 1 | S | 16 | Tight cord, smoking | Epilepsy, sleep disturbances | TMEM194A (de novo) | RNA binding |
| 4349 | P021 | M | 39 | SpH | 2 | S | 4 | tight cord, possible foetal restriction (reduced movement late in pregnancy), IUGR | Seizures, day 2. Mild ID, speech dyspraxia, acute left MCA territory infarct |  | development, sterol biosynthesis, morphogenesis |
| 4350 | P022 | M | 38 | Q | 4 | S | 2 | IUGR | Severe neonatal encephalopathy, seizures from birth |  | sodium ion transport, ATPase activity, structural constituent of cytoskeleton |
| 4437 | P023 | F | 39 | SpQ | 4-5 | S | 5 | cholestasis | Moderate ID, microcephaly, epilepsy, generalised polymicrogyria, non-verbal | CGH array dup 2q37.3 of unknown significance, supported by RNA seq, D2HGDH 4.26, ING5 2.65, ATG4B 3.02, CAPN10 2.36 | splicesome complex, nuclear pore, RNA metabolism |
| 4438 | P024 | F | 40 | SpH | 1 | S | 15 | high blood pressure, tight cord | Mild ID, hydrocephaly, global, DD, focal epilepsy, behaviour and sleep problems |  | intra-golgi transport, hemopoiesis, development |
| 4569 | P026 | F | 39 | SpD |  | S | 15 | Anaemia, IUGR, Flu 21 weeks + | Mild ID, speech dyspraxia, convergent strabismus. Ridging metopic suture, mild trigonocephaly. Severe behaviour problems. | KDM5C (de novo), CENPF (de novo) | programmed cell death, transcription factor binding, defense response, signal transduction |
| 4570 | P027 | M | 29 | SpQ | 4 | S | 14 | Pre-eclampsia, IUGR | Mild ID, respiratory distress syndrome. | PAK4 (de novo)  z-score = -2.50 | innate immune response, inflammatory response, cell-cell signalling |
| 4577 | P028 | F | 41 | SpH | 1 | S | 16 | Tight cord, smoking | Epilepsy, problematic fatigue |  | cytosolic ribosome, small monomeric GTPase activity |
| 4590 | P029 | M | 34 | H | 4 | S | 11 | Placental abruption at 34 weeks | Global DD, severe ID, generalized tonic clonic and focal seizures, left divergent strabismus, turrencephaly. Suggestion of mild hypotelorism and slight midline ridging of the forehead, orbital roofs rise slightly more steeply than usual | Mosaic chromosome ring 22, De novo 46,XY,r(22)(p11.2q13.3 [30]/46,XY [90], SSPO (de novo), **NAGLU** z-score=-4.02, charcot-marie-tooth-disease, MPSIIIB. | morphogenesis, organogenesis, development, cytoskeleton, microtubule |
| 4591 | P030 | M | 37 | SpQ | 5 | S | 18 | IUGR, Cold/flu 21+ weeks | Epilepsy, cataracts, cortical visual impairment, no speech, scaphocephaly | TNKS1BP1 (de novo), CTDSPL (de novo) | signal transduction, regulation of cell shape, cell communication, cellular morphogenesis |
| 4630 | P031 | M | 28 | SpH |  | S | 2 | Preeclampsia, Gastric infection + cold/flu 1-20 weeks, IUGR | Borderline ID, focal epilepsy, Ponto-cerebellar hypoplasia with partial aplastic corpus callosum | DNAH17 (CH), POTEC (CH), ARMCX2 (X-linked) | cytoskeleton, M phase, cell communication |
| 4631 | P032 | M | 27 | SpH | 2 | S | 4 | Herpes 21+ weeks. Bleeding at 25 weeks, haemorrhage at 27 weeks. History of premature birth (previous 29 weeks). | Patent foramen ovale, patent ductus arteriosus | LOC388946  (de novo) | carbon-carbon lyase activity, chaperone activity |
| 4632 | P033 | M | 37 | SpQ | 4 | S | 6 | Asthma | Severe ID, epilepsy. | duplication chr 8p, significance not clear at time of diagnosis, AGAP1 (de novo)  z-score=-2.13 | cell communication, signal transduction, apoptosis |
| 4633 | P034 | M | 40 | SpH | 1 | S | 10 | Home birth, Cold/flu 21+ weeks | Epilepsy, visual impairment |  | RNA processing, spliceosome complex |
| 4646 | P035 | F | 27 | SpH | 2 | S | 12 | Severe allergic reaction to bee-sting at 20 weeks, preeclampsia, hay fever. Cold/flu 1-20 weeks, IUGR | Moderate ID, epilepsy, divergent squint, thyroglossal cyst removed, clinically thyroid agenesis, Sleep apnoea. Recurrent ear infections. Growth and feeding problems in history. Deterioration of motor function and bladder control at age 5 yr. | COL4A1 (de novo) | lipid metabolism, golgi apparatus |
| 4648 | P036 | F | 41 | SpH | 1 | S | 8 | High blood pressure, IUGR | ASD, learning difficulties, thyroid agenesis (congenital hypothyroidism), carrier of 5p11.2 duplication, mildly abnormal EEG due to focal slowing, suggestive of underlying focal cortical dysfunction | APOB (CH) | mitochondrion, proteasome complex |
| 4651 | P037 | F | 25 | H |  | S | 23 |  | Trigoncephaly, left frontal haemorrhage in the newborn period | LENG8 (de novo) | sterol metabolism, translation elongation factor activity, transcription from polII promoter |
| 4681 | P038 | M | 32 | SpD |  | Tw | 32 |  | Primary hypertension, anaemia, mild right hydronephrosis due to single calcified opacity. No brain imaging. | SLA (de novo), ZDHHC9 (X-linked), OFD1 (X-linked), **CHD8** z-score=-4.48, susceptibility to autism | ribonucleoprotein complex, mitochondrion |
| 4683 | P025 | M | 40 | SpH | 1 | S | 6 | Cold/flu 1-20 weeks | Severe sleep disturbances. Anxiety issues. Behaviour problems | MAOB (X-linked), HSPG2 (CH) | small GTPase mediated signal transduction, cell communication, spindle pole, microtubule organising centre |
| 4684 | P039 | M | 38 | SpD | 2 | S | 8 | Smoking, Cold/flu within 1 week of birth | Likely moderate generalised delay in myelination, however leukodystrophy not ruled out | CNDP2 (de novo), **CD99L2** (X-linked) z-score = -4.20 | defense response, response to biotic stimulus, nitric oxide mediated signal transduction |
| 4726 | P040 | M | 38 | SpQ | 5 | S | 13 | High blood pressure, cord caught, hospital admission for chest infection 36 weeks, tight cord | Severe ID, epilepsy, sleep disturbances, microcephaly, global DD. Hepatomegally, both kidneys malrotated. | MTMR1 (X-linked) | receptor activity, signal transducer activity, cell communication |
| 4727 | P041 | F | 40 | SpH | 3 | S | 20 | Ruptured ovarian cyst 8 weeks | Large left porencephalic cyst in the territory supplied by the middle cerebral artery, epilepsy, intellectual decline, mild/moderate ID, homonymous hemianopia. |  | carbohydrate catabolism, energy derivation by oxidation of organic compounds |
| 4740 | P042 | F | 32 | SpH |  | S | 24 | High blood pressure, signs of preeclampsia, IUGR | Mild ID, behaviour problems. Focal seizures, abnormal EEGs. |  | cation transporter activity, hydrogen ion transporter activity, carbohydrate metabolism, aminoglycan metabolism |
| 4744 | P043 | F | 41 | SpH | 2 | S | 12 | Cold/flu + fever 21+ weeks | Mild ID, smaller left brain in general, heart murmur. | SCN8A (de novo), ATP11B (de novo), SIPA1L1 (de novo) | lipid metabolism, sterol metabolism |
| 4745 | P044 | F | 30 | T | 3 | S | 2 | Head injury in utero at 29 weeks due to accident, morphine given, anaemia | Hydrocephalus, porencephalic cyst secondary to infarct | WIPI2 (de novo), **NGDN** z-score = -4.56, translational regulatory protein important during development of the vertebrate nervous system | negative regulation of transcription from Pol II promoter, epidermal differentiation |
| 4746 | P045 | F | 39 | SpH | 1 | S | 6 | Gastric infection 21+ weeks, tight cord | Deep right sided brain lesion | ABLIM2 (de novo) | structural constituent of ribosome, hydrogen ion transporter activity, mitochondrion |
| 4747 | P046 | F | 38 | Dys/D | 2 | S | 2 | High blood pressure, Gastric infection 21+ weeks | Seizures from neonate, dyspraxia, likely infarction involving the frontal and parietal lobes bilaterally |  | mitochondrion, hydrogen ion transporter activity, endoplasmic reticulum, monovalent inorganic cation transporter activity |
| 4748 | P047 | M | 29 | SpQ | 5 | Tw | 10 | Asthma, waters broke 27 weeks, Cold/flu 21+ weeks, IUGR | Microcephaly |  | mitochondrion, hydrogen ion transporter activity, monovalent inorganic cation transporter activity, mitochondrial membrane |
| 4793 | P049 | M | 35 | SpH | 1 | S | 8 | Cold/flu 21+ weeks, IUGR | Sub-ependymal heterotopia suggesting minor neuronal migration disorder | HSPG2 (CH), SREBF1 (de novo) z-score = -2.68, ANKRD44 (de novo) z-score = -2.70 | lipid metabolism, alcohol metabolism, sterol biosynthesis |
| 4895 | P050 | M | 35 | Asym/SpD |  | S | 4 | Mother epilepsy, high blood pressure, bowel obstruction treated by laproscopy, secondary to endometriosis, Gastric infection 1-20 weeks | Epilepsy |  | transferase activity, transferring phosphorus-containing groups, cell cycle |
| 4979 | P051 | M | 39 | SpH | 2 | S | 10 |  | Mild dilatation of anterior horn of right lateral ventricle, consistent with porencephalic cyst |  | electron transport, NLS-bearing substrate-nucleus import, response to abiotic stimulus, transporter activity, metabolism |
| 4984 | P052 | F | 38 | SpQ | 5 | S | 15 | Alcohol 1-2 per week, hospitalised for asthma 16+ weeks, severe fever from viral infection, required hospitalisation and rehydration 21+ weeks | Mild ID, epilepsy, progressive leukodystrophy in family, 2 siblings deceased. Gradual but progressive decline neurodevelopmentally. Past history also reveals gastrostomy, nissens’s funcoplication, swallowing delay, possible bleeding disorder | POLG (CH) | intracellular signaling cascade, peptidase activity, protein transporter activity |
| 5113 | P053 | M | 36 | SpQ | 4-5 | S | 9 | Gestational diabetes, cervical suture for incompetent cervix, Cold/flu 1-20 weeks | Persistent hypoglycaemia during first week, persistent thrombocytopenia, mild ID, ASD. | 18p11.21 dup, 5q21.1 del, 22q13.33 dup of unknown significance, **HUWE1** (X-linked, 2 variants), z-score = -3.67, E3 ubiquitin ligase, X-linked mental retardation, syndromic, **CHD8,** z-score = -5.18 | structural constituent of ribosome, mitochondrion |
| 5114 | P054 | M | 40 | SpD | 2 | S | 6 |  |  | SPAST (CH, one splice site mutation appearing to cause exon skipping) | endoplasmic reticulum, glycoprotein metabolism, carbohydrate metabolism |
| 5115 | P055 | F | 29 | H | 1 | Tw | 6 |  |  | **CHD8**, z-score= -5.06 | Structural constituent of ribosome, macromolecule biosynthesis, mitochondrion |
| 5116 | P056 | M | 36 | SpQ | 3 | S | 10 | IUGR, smoking | DD |  | translation regulator activity, proteasome regulatory particle, mitochondrion |
| 5117 | P057 | M | 29 | H |  | S | 10 | Anaemia | Cortical visual impairment. Grade III IVH. Developed meningitis with significant cystic cerebral degeneration and obstructive hydrocephalus. At 6 weeks sudden deterioration with ultrasound brain anomalies. Multiple porencephalic cysts. |  | [hydrogen ion transporter activity, monovalent inorganic cation transporter activity, mitochondrion](http://www2.ebi.ac.uk/ego/QuickGO?mode=display&entry=GO%3A0015078) |
| 5118 | P058 | M | 38 | Q | 4 | S | 3 |  | Mild ID, Epilepsy, DD, Congenital CMV infection, generalized polymicrogyria, Several febrile seizures | PTPN14 (de novo), INHBB (de novo), **MMADHC** (stopgain, inherited from Mother) z-score = -4.174, Homocystinuria and Methylmalonic Aciduria, causes neurologic abnormalities, including global developmental delay, spastic ataxia, difficulties with speech, eye contact | cell growth and/or maintenance, transcription regulator activity |
| 5158 | P059 | M | 25 | D | 3 | S | 15 | Major bleeding during labour | mild ID, ASD, severe language disorder characterised by difficulties understanding and using language, vision impaired |  | intracellular protein transport, RNA polymerase complex, phosphotransferase activity, alcohol group as acceptor, diacylglycerol kinase activity |
| 5239 | P060 | F | 40 | H | 3-4 | S | 3 | Recreational drugs, smoking | Epilepsy, macrocephaly, significant speech, language and cognitive delay noted, Developmental brain abnormality: bilateral frontal polymicrogyria | Grandmother and aunt CP, KANK1, PLA2G6 (inheritance unknown)+ | Metallo-endopeptidase activity, hematopoietin interferon-class (D200-domain) cytokine receptor activity, proteolysis and peptidolysis |
| 5240 | P061 | M | 27 | D | 4 | S | 3 | Major bleeding during labour, smoking | Significant visual impairment. Antepartum IVH and intracerebral haemorrhage (Grade IV) secondary to placenta praevia, had sign early lung disease and developed hydrocephalus, ventricular peritoneal shunt to address this. Renal cyst which resolved. | BRWD3 (X-linked) | [nucleobase, nucleoside, nucleotide kinase activity, kinase activity, induction of apoptosis](http://www2.ebi.ac.uk/ego/QuickGO?mode=display&entry=GO%3A0019205) |
| 5241 | P062 | F | 29 | SpD | 4 | S | 5 |  |  |  | ATP binding, metabolism, antiviral response protein activity |
| 5242 | P063 | F | 42 | Dys/T | 1-2 | S | 6 |  | Language and speech delay. Nonspecific periventricular and bilateral internal capsule white matter abnormality. |  | metabolism, DNA repair, mitotic cell cycle, response to endogenous stimulus |
| 5243 | P064 | M | 28 | SpT | 2 | S | 6 |  | IVH and hydrocephalus. Moderate ID, epilepsy, vision impairment. Appearance consistent with marked periventricular leucomalacia with post-ischaemic encephalopathic cortical defects forming a type of schizencephaly with hypoplasia of the corpus callosum but without true agenesis |  | DNA repair, response to endogenous stimulus, response to DNA damage stimulus |
| 5296 | P065 | F | 32 | H | 1 | S | 12 | Fever within 1 week of birth, IUGR | Grade II IVH. Squint operation. |  | regulation of transcription, DNA-dependent, excitatory extracellular ligand-gated ion channel activity, cell-cell signalling, nicotinic acetylcholine-activated cation-selective channel activity |
| 5382 | P066 | M | 24 | AD |  | S | 20 | Smoking, UTI, infection/fever during labour, Herpes + GBS 21 weeks+ | Bronchopulmonary dysplasia, ASD, Congenital squint and nystagmus and severe myopia. Chronic bronchitis |  | [isomerase activity, MAP kinase phosphatase activity, cell organization and biogenesis, prostaglandin biosynthesis](http://www2.ebi.ac.uk/ego/QuickGO?mode=display&entry=GO%3A0016853) |
| 5461 | P067 | F | 28 | D | 3 | S | 2 | Major bleeding during labour, Herpes 1-20 weeks and 21+ weeks + |  | UBXN7 ( de novo) | [regulation of transcription, DNA-dependent, potassium ion transport](http://www2.ebi.ac.uk/ego/QuickGO?mode=display&entry=GO%3A0006355) |
| 5494 | P068 | M | 39 | H | 2 | S | 6 | Alcohol 1-2 per week, admitted 2x for bowel pain in last 4 weeks, Cold/flu + fever 21+ weeks | Mild scaphocephaly consistent with intrauterine moulding. Hypoglycaemia. Possible left septal hypertrophy. Possible right ventricular hypertrophy. Borderline ECG |  | [transcription, polysaccharide metabolism, synaptic transmission](http://www2.ebi.ac.uk/ego/QuickGO?mode=display&entry=GO%3A0006350) |
| 5521 | P069 | F | 38 | H | 1 | S | 5 |  |  |  | [antimicrobial humoral response (sensu Vertebrata), calmodulin binding, cell communication, determination of left/right symmetry](http://www2.ebi.ac.uk/ego/QuickGO?mode=display&entry=GO%3A0019735) |
| 5522 | P070 | M | 34 | H | 1 | Tw | 5 | IUGR | Hydrocephalus | ARSA, KANK1 (inheritance unknown) | [regulation of transcription, DNA-dependent, extracellular matrix, polysaccharide metabolism, aromatic compound biosynthesis, synaptosome](http://www2.ebi.ac.uk/ego/QuickGO?mode=display&entry=GO%3A0006355) |
| 5523 | P071 | M | 40 | SpH | 1 | S | 9 | Cold/flu + fever 21+ weeks | Mild ID, epilepsy, focal and partial seizures without loss of consciousness | **IDH3B** z-score = -4.57, catalyses the oxidative decarboxylation of isocitrate into alpha-ketoglutarate | [cytoskeleton, carboxylic acid metabolism, cytoplasm organization and biogenesis, microtubule cytoskeleton, actin binding](http://www2.ebi.ac.uk/ego/QuickGO?mode=display&entry=GO%3A0005856) |
| 5524 | P072 | M | 40 | SpQ | 4 | S | 10 | Smoking | Localised porencephaly |  | isomerase activity, receptor binding, nonselective vesicle transport, cation channel activity, excitatory extracellular ligand-gated ion channel activity, apoptosis inhibitor activity |
| 5546 | P073 | M | 40 | SpQ | 2 | S | 13 |  | Seizures as neonate. |  | [signal transducer activity, defense response, response to biotic stimulus, immune response, cell communication](http://www2.ebi.ac.uk/ego/QuickGO?mode=display&entry=GO%3A0004871) |
| 5577 | P074 | F | 37 | SpD | 2-3 | S | 8 |  | Global DD, epilepsy | ALDH3A2 (CH) | [ribosome, extracellular matrix, growth factor activity, cytokine activity](http://www2.ebi.ac.uk/ego/QuickGO?mode=display&entry=GO%3A0005830) |
| 6085 | P075 | F | 34 | Dys/SpQ | 3 | S | 3 | Smoking, Alcohol 1-2 per week, IUGR | Previous resolved renal calculi, right posterior frontal haematoma. |  | extracellular matrix, transcription factor activity, negative regulation of cell proliferation, transcription factor complex, transition metal ion binding |
| 6086 | P076 | F | 37 | SpQ | 2 | S | 6 | IUGR, Gastric infection 1-20 weeks | Mild unilateral cerebral ventricular dilatation at 19 weeks gestation. Amniocentesis; normal. Growth slowed down significantly from 30 weeks. Developed moderate hyaline membrane disease. Microcephaly, ID, ASD, poor sleeping from early in life. Strabisumus. Mildly dysmorphic features, multiple wrinkles under eyes, tin upper lip with a cupid’s bow structure. |  | structural constituent of ribosome, translation elongation factor activity, metabolism, cytoplasm organization and biogenesis |
| 6087 | P077 | F | 28 | SpH | 1 | S | 8 |  | Moderately severe respiratory distress syndrome, and sepsis. Agenesis of septum pellucidum, bilateral close lip schizencephaly. |  | structural constituent of ribosome, macromolecule biosynthesis, metabolism, DNA packaging, aerobic respiration |
| 6123 | P078 | M | 29 | SpD | 3 | S | 2 | Tooth infection 21+ weeks | Sensory processing disorder. Prominent periventricular high signal which would suggest previous white matter injury and gliosis. | Great Uncle (paternal) CP | structural constituent of ribosome, eukaryotic 48S initiation complex, inflammatory response, innate immune response |
| 6124 | P079 | M | 40 | SpH | 1 | S | 4 | IUGR | Focal encephalomalacia/ periventricular leucomalacia within posterior aspect of right corona radiata at junction of the right parietal and temporal lobes. |  | nucleic acid binding, antimicrobial humoral response, hydrolase activity, acting on glycosyl bonds, transcription factor activity, endoplasmic reticulum |
| 6125 | P080 | M | 27 | Dys/SpQ | 5 | S | 18 |  | Inguinal hernia, microcephaly. Mild periventricular white matter loss | ARHGAP21 (CH) | transcription factor activity, MAP kinase phosphatase activity, isomerase activity, nonselective vesicle transport, cytokine activity. |
| 6138 | P081 | F | 36 | H | 3 | S | 2 | Smoking | Schizencephaly in the right frontal parietal region with associated mild colpocephaly affecting the body/atrium of the right lateral ventricle, 2^nd^ cousin CP | 2nd cousin CP, TTN (CH) | membrane, muscle development, lytic vacuole, lysosome, development |
| 6139 | P082 | F | 38 | Q | 4 | S | 3 | IUGR | Moderate ID, hearing & vision loss, microcephaly, swallowing issues, calcification of basal ganglia, cystic areas bilaterally in occipital white matter. Diffuse polymicrogyria, consistent with congenital infection. CMV diagnosed by blood test at 1 year old. |  | response to external stimulus, defense response, sensory perception, signal transducer activity, cell-cell adhesion |
| 6140 | P083 | M | 25 | Dys/SpD | 3 | S | 4 | Placental abruption 25 weeks | Persistent ductus arteriosus, feeding difficulties, GORD, failure to thrive, squint, speech delay, lumbar scoliosis, DD. | IQSEC2 (X-linked), SLITRK2 (X-linked), **VRK3** z-score = -5.87, suppresses ERK activity by promoting phosphatase activity of DUSP3 | response to external stimulus, defense response, transcription factor activity, di-, tri-valent inorganic cation transport, calcium ion transport |
| 6141 | P084 | F | 40 | DyskD | 3 | S | 11 |  | Multi organ failure, seizures and need for ventilation at birth. Epilepsy. |  | Golgi apparatus, endomembrane system, phosphoric ester hydrolase activity, carbohydrate metabolism, cell communication |
| 6293 | P096 | M | 30 | D | 3 | S | 1 | Major bleeding in labour, UTI 0-20 weeks and 21 weeks +, IUGR | Single focal seizures with secondary generalisation, Motor and expressive speech DD at 4 yrs of age. Social skills delay. Sleep disturbances. | Maternal aunt CP | DNA-directed RNA polymerase II, core complex, RNA splicing, calmodulin binding, DNA replication |
| 6294 | P097 | M | 30 | SpD | 3 | S | 2 | Alcohol 1-2 per week, placenta praevia, Gastric infection 1-20 weeks |  |  | small nuclear ribonucleoprotein complex, G-protein signaling, coupled to IP3 second messenger (phospholipase C activating), transcription factor complex, intracellular signaling cascade |
| 6295 | P098 | F | 28 | SpD | 2 | S | 2 | Alcohol 1-2 per week |  | Novel deletion Chr10, Supported by RNA seq DNAJB12 z-score = -4.40, ECD z-score = -4.32, MICU1 z-score = -4.06 | cell communication, cytosolic large ribosomal subunit, signal transduction, immune response |
| 6296 | P099 | M | 32 | SpD | 3 | Tw | 9 |  | Hyaline membrane disease, Strabismus, mild optic nerve dysfunction and inferior visual field defect. | Cousin with CP | defense response, immune response |
| 6477 | P101 | M | 40 | SpQ | 4 | S | 13 | Threatened miscarriage (history early miscarriage), intra-venous gamma-globulin, IUGR | Dysphagia and dysarthria, severe dystonia, ID, no speech, understands conversation, communicates well with sign language and computer. Fronto-parietal temporal dysplasia, pachygyria appearance, polymicrogyria. Wrist in flexion and ulnar deviation. Anxiety problems, easily agitated, gagging. Parents first cousins. | NGFR (de novo) | intercellular junction, apicolateral plasma membrane, hydrolase activity, acting on glycosyl bonds, steroid biosynthesis, M phase of mitotic cell cycle |
| 6478 | P102 | M | 39 | H | 1 | S | 17 | Warfarin injection at 7mths-9ths due to heart value replacement | Epilepsy, features of left cerebral cortical dysplasia particularly involving the frontal and parietal lobes, appears to be pachygyria/ polymicrogyria spectrum in keeping with a neuronal migration disorder. | ZMYM3 (X-linked), MAML3 (CH) z-score = 5.03 | proteasome complex, G-protein coupled receptor protein signaling pathway, kinase activity, rhythmic behavior, monooxygenase activity |
| 6479 | P103 | M | 36 | SpH | 1 | S | 18 |  | Epilepsy, ASD, speech problems, ID, significant behaviour problems, attention seeking behaviour, interruptive and manipulative at a young age. Small peri-membranous ventricular septal defect at 12 months of age, spontaneously closed. Right inguinal hernia. | STAB2 (de novo), **PAK4** z-score = -3.72, regulates cell morphology, cytoskeletal organization, and cell proliferation and migration **TRAF7** z-score = -6.19, signal transducer for TNF receptor superfamily **MED24** z-score = -4.37, component of the Mediator complex, a coactivator involved in the regulated transcription of nearly all RNA polymerase II-dependent genes MECP2 z-score = -3.22 | Too many genes to analyse |
| 6480 | P100 | F | 40 | SpD | 2 | S | 6 | Smoking |  | **TRAF7** z-score = -4.59, signal transducer for TNF receptor superfamily | DNA binding, transcription, metabolism, sensory perception, hearing |
| 6493 | P104 | M | 40 | H |  | S | 2 | IUGR | Congenital hyper-insulinaemia, severe hypoglycaemia, severe thrombocytopenia, basal ganglia haemorrhages, hydrocephalus, accommodative estropia |  | cell junction, spliceosome complex, antiviral response protein activity |
| 6494 | P105 | F | 38 | Dys/SpQ | 3 | S | 2 | Anaemia, IUGR | ID, epilepsy,  neonatal hypoglycaemia and subsequent seizures. | JHDM1D (de novo), PHF17 (de novo) | Response to biotic stimulus, immune response, oxidoreductase activity, acting on the CH-OH group of donors, NAD or NADP as acceptor, mitochondrion |
| 6495 | P106 | M | 39 | SpH | 1 | S | 4 |  | Bilateral femoral anteversion | ZNF160 (de novo) | RNA ligase activity, amino acid transporter activity, sterol metabolism, electrochemical potential-driven transporter activity, G1 phase of mitotic cell cycle |
| 6496 | P107 | F | 23 | H |  | Tw | 4 | Smoking, infection in uterus, major bleeding, Fever 21+ weeks and within 1 week of birth, IUGR | Epilepsy, vision problems, DD. Left Grade IV IVH, porencephaly, hydrocephalus, marked left hemiatrophy and encephalomalacia. | ACADM (inheritance unknown) | Response to biotic stimulus, immune response, regulation of cell proliferation, cell communication, post-Golgi transport |
| 6497 | P108 | M | 26 | SpD | 4 | S | 11 | Preeclampsia, IUGR | Monocular visual loss, ID |  | proteolysis and peptidolysis, cell communication, neutral amino acid transporter activity, cell adhesion, steroid hormone receptor activity |
| 6652 | P401 | F | 35 | H |  | Tw | 6 |  | Twin 1 |  | kinase activity |
| 6653 | P402 | M | 26 | SpD |  | Tw | 6 |  | Twin 2, Epilepsy | Second cousin CP, **PEX13** z-score = -4.39, peroxisomal membrane protein | cytoskeletal protein binding, actin binding |
| 6654 | P403 | M | 35 | SpD | 1 | S | 7 | Smoking, IUGR, Gastric, urea plasma infection 21+ weeks | Strabismus | TMEM150A (de novo) | glycoprotein metabolism, sulfur amino acid metabolism, microtubule cytoskeleton |
| 6655 | P404 | F | 29 | SpD | 1 | Trip | 7 |  | Triplet 3 |  | mitochondrion organization and biogenesis, cis-trans isomerase activity, mitochondrial transport |
| 6656 | P405 | M | 30 | D | 1 | S | 8 |  |  | CDH26 (de novo), ENOX2 (X-linked) | Acyltransferase activity, metal ion binding, amine catabolism, calcium ion binding, blood coagulation |
| 6673 | P406 | F | 30 | H |  |  | 15 |  |  | TUBA3D (de novo) | nuclear division, M phase, immune response |
| 6674 | P407 | M | 29 | H |  | Tw | 4 |  | Twin 1 |  | oxidoreductase activity, protein kinase regulator activity, electron transporter activity, immune response |
| 6675 | P408 | M | 36 | H |  | S | 5 | Smoking, tight cord, fever in labour |  | **LCMT1** z-score =  -4.39, catalyzes the methylation of the carboxyl group of the C-terminal leucine residue (leu309) of the catalytic subunit of protein phosphatase-2A | membrane, one-carbon compound metabolism, ion transporter activity |
| 6676 | P409 | M | 37 | H | 2 | S | 7 | Smoking, high blood pressure, diabetes, asthma, Cold/flu 1-20 weeks, antidepressants during pregnancy (Effexa) | ASD, ADHD | MCCC1 (inheritance unknown), **WDR74** (stop-gain, inheritance unknown) z-score = -4.16, putative novel regulatory protein of the MTR4-exsosome complex | transmembrane receptor activity, G-protein coupled receptor activity, metal ion transport, cell communication, cation transport |
| 6677 | P410 | F | 39 | H | 1 | S | 8 | Trauma - assault, kicked in lower back, IUGR | Epilepsy |  | microbody, peroxisome, response to stress |
| 6680 | P411 | F | 27 | SpQ | 5 | S | 13 | Sinusitis, major bleeding in labour, smoking, IUGR |  | KIAA0415 (CH) | transport, hydrolase activity\, acting on acid anhydrides\, catalyzing transmembrane movement of substances, ATPase activity\, coupled to transmembrane movement of substances |
| 6682 | P109 | M | 35 | H | 1 | S | 9 | High blood pressure, smoking, IUGR | Epilepsy, significant learning difficulties, obesity, left homonymous hemianopia |  | mRNA-nucleus export, mitochondrion, nucleocytoplasmic transporter activity, transferase activity, transferring phosphorus-containing groups, electron transport |
| 6683 | P110 | M | 29 | H | 2 | Tw | 3 | IUGR, High blood pressure, gestational diabetes, other twin deceased 28 weeks after reverse flow on doppler at 24 weeks | Twin 2, convergent squint, likely ASD, grade 4 IVH |  | response to DNA damage stimulus, response to stress, transketolase activity |
| 6684 | P111 | M | 41 | SpQ | 3 | S | 2 |  | Mild squint and hypermetropia. Thrombocytopenia. | EIF4E2 (de novo), SACS (CH) | fatty acid metabolism, lipid metabolism, behavior, neurogenesis |
| 6685 | P412 | M | 30 | SpQ |  | S | 18 | Smoking, IUGR |  | PLA2G6 (inheritance unknown) | none |
| 6700 | P413 | M | 37 | D |  |  | 3 | Car accident 2 weeks before birth, Cold/flu 21+ weeks |  | Mother and proband hereditary spastic paraplegia | signal transducer activity, cell communication, cell surface receptor linked signal transduction, immune response, Wnt receptor signaling pathway |
| 6701 | P414 | M | 40 | H | 1 | S | 10 | Alcohol 1-2 per week, Antidepressants during pregnancy, UTI | Epilepsy, von Willebrand disease | MAN2A1 (de novo) | mitochondrion, embryogenesis and morphogenesis, di-, tri-valent inorganic cation transporter activity |
| 6702 | P415 | F | 39 | SpD | 2 | S | 12 | Herpes within 1 week after birth | Mild ID, DD | Brother with severe ASD. | nuclear division, M phase, mitosis, cell communication, cytoskeleton organization and biogenesis |
| 6703 | P416 | M | NA | SpD | 4 | Tw | 12 |  | Twin 2 | SPAST (inheritance unknown) | mitochondrion, lipid biosynthesis, mitochondrial matrix, cholesterol metabolism, oxidoreductase activity |
| 6717 | P417 | M | 39 | H |  | S | 4 | Diabetes | Right temporal lobe infarct | Sister with epilepsy, global DD and diplegic spasticity, **MED24** z-score = -4.32, component of the Mediator complex, a coactivator involved in the regulated transcription of nearly all RNA polymerase II-dependent genes | response to stress, protein kinase cascade, transcription regulator activity, immune response, intracellular signaling cascade |
| 6718 | P418 | F | 36 | H |  | S | 6 | Smoking, alcohol 3-4 per week, recreational drugs, placenta praevia, UTI, electric shock, spider bite |  | UROD z-score = -4.60 | protein-tyrosine kinase activity, ATP binding, protein serine/threonine kinase activity, regulation of transcription, DNA-dependent |
| 6719 | P419 | F | 40 | D | 3 | S | 9 | Alcohol 1-2 per week, major bleeding in labour, tight cord, Fever within 1 week after birth, IUGR | Strabismus | **KIDINS220** z-score = -4.30, schizophrenia risk, neurotrophin signalling, regulator of neuronal and cardiovascular development | DNA replication and chromosome cycle, DNA replication, S phase of mitotic cell cycle |
| 6720 | P420 | M | 38 | H |  | S | 10 |  | Epilepsy, stroke 3^rd^ trimester |  | cell communication, Golgi apparatus, signal transduction, defense response, intracellular signaling cascade, COPII vesicle coat, circulation |
| 6749 | P422 | F | 28 | D |  | S | 6 | Underactive thyroid in pregnancy | Dysplastic kidney, ureter in incorrect position |  | carbohydrate kinase activity, signal transducer activity, transcription cofactor activity, phosphofructokinase activity, glycolysis |
| 6829 | P112 | F | 41 | H | 4 | S | 9 |  | Mild ID, short stature, ASD. Hypotonia in neonatal period. Large teratoma at 13 months of age. Signs of obstructive hydrocephalus and DD. Since surgical removal, extensive history of hospital admissions due to shunt infections/ movement. Seizures started post-surgery. First tonic-clonic, now complex-partial type. Also agitation, extreme anxiety, OCD, psychosis, tantrums, sensory impairments. Hemianopsia. Left sided hearing impairment, large vestibular duct syndrome |  | cell communication, intracellular signaling cascade, cell adhesion, development, cortical actin cytoskeleton, defense response, neurogenesis |
| 6868 | P423 | F | 27 | SpD |  | S | 3 | History of premature labour, sibling born at 25 weeks |  |  | actin binding, cell surface receptor linked signal transduction, Wnt receptor signaling pathway |
| 6869 | P424 | M | 38 | D |  | S | 4 | Smoking | Moderate ID, global DD | L1CAM (X-linked) | amino acid and derivative metabolism, amine metabolism, RNA modification |
| 6870 | P425 | M | 38 | H |  | S | 11 | Gastric infection 1-20 weeks | Epilepsy, stroke in utero. | Chr16p11.2 deletion supported by RNA seq data, **KCTD13** z-score = -4.71, major driver for the neurodevelopmental phenotypes associated with the 16p11.2 CNV - including autism and microcephaly | hexose metabolism, glycolysis, energy pathways, cell-matrix adhesion |
| 6927 | P426 | F | 33 | H | 2 | Tw | 4 | Preeclampsia 29-30 weeks. Mother 3 miscarriages since this pregnancy | Twin 2 (identical), epilepsy, genital anomaly. Sibling twin has congenital foot deformity and cranial stenosis. |  | cell surface receptor linked signal transduction, G-protein coupled receptor protein signaling pathway |
| 6928 | P427 | M | 38 | H | 2 | S | 6 | Cold/flu 21+ weeks | Stroke in utero. |  | carrier activity, ATP-binding cassette (ABC) transporter activity, inorganic anion transporter activity, electrochemical potential-driven transporter activity |
| 6929 | P428 | M | 29 | SpD | 2 | Trip | 9 |  | Triplet 2, Mild ID, chronic migraine | UBQLN3 (de novo) | sterol biosynthesis, lipid biosynthesis, isoprenoid metabolism, peroxisome |
| 6977 | P429 | M | 38 | H |  | Tw | 8 |  | Epilepsy | Maternal uncle CP, **STK16** (stop gain, inherited from Mother) z-score = -3.65, involved in secretory pathway. Overexpression leads to disorganization of the Golgi apparatus into vesicular structures. | clathrin vesicle coat, Golgi apparatus |
| 6978 | P430 | F | 37 | Asym/D | 3 | S | 12 | Thrombo-cytopenia, Smoking | Epilepsy, non-verbal, DD | **DIP2B** z-score = -4.39, FRA12A mental retardation associated with reduced expression due to repeat expansion and methylation, with seizures and behavioural problems in some cases | nuclear organization and biogenesis, cell proliferation, synaptic vesicle, clathrin-coated vesicle, histogenesis and organogenesis |
| 6988 | P431 | F | 40 | H |  | S | 12 | Alcohol 1-2 per week, thyroid medication, Smoking, IUGR |  |  | RNA binding, protein biosynthesis, mitotic cell cycle |
| 7016 | P432 | F | 41 | D | 2 | S | 8 | Low blood pressure | ASD, toe walker, DD | 2p25.3 deletion, supported by RNA seq, **TMEM18** z-score = -4.57, involved in the migratory response of neural precursors, **ACP1** z-score = -6.30 | extracellular matrix, cell adhesion receptor activity, cell motility, Golgi apparatus, cytoskeleton |
| 7017 | P433 | F | 37 | Q | 5 | S | 5 | No doctor at birth, cord around neck, Gastric infection and herpes 21+ weeks | Epilepsy with seizure onset as neonate |  | proton-transporting two-sector ATPase complex, cytoskeleton, actin binding, muscle contraction, cell adhesion molecule activity |
| 7018 | P434 | F | 24 | H |  | S | 9 | UTI, infection/ fever during labour (*E. coli* septicaemia), history of pre-term birth |  |  | heterocycle metabolism, development, mitochondrial inner membrane, G-protein signalling, adenylate cyclase activating pathway, cell cycle arrest, regulation of cell shape |
| 7028 | P435 | M | 39 | D | 1 | S | 11 | Asthma, minor trauma, Gastric infection 21+ weeks, Father genital herpes but no known exposure, IUGR | Dyscalculia, ADHD, dyslexia, DD. | ZNF697 (de novo), IGFBP1 (X-linked) | ubiquitin thiolesterase activity, transferase activity, transferring pentosyl groups, lipid metabolism, muscle development |
| 7055 | P436 | F | 30 | SpD | 3 | Tw | 4 | IVF, Diabetes, IUGR | Twin 2 | SREK1 (de novo), GANC (de novo) | transcription factor binding, Golgi apparatus, cytoplasmic vesicle, DNA damage response, signal transduction resulting in cell cycle arrest |
| 7056 | P437 | M | 28 | Dys/H | 5 | S | 9 | Major bleeding during labour, Herpes 1-20 weeks and 21+ weeks | Epilepsy, bilateral profound deafness |  | lipid metabolism, isoprenoid biosynthesis, negative regulation of cell cycle, muscle development |
| 7057 | P438 | M | 33 | H | 1 | S | 12 | Fibromyalgia | Mild ID, Ventriculomegaly, degeneration of thalamus and the corpus callosum. | L1CAM (de novo) | extracellular matrix structural constituent, collagen type IV, regulation of transcription, signal complex formation |
| 7058 | P439 | M | 29 | Asym/D |  | Tw | 14 | Fibromyalgia, IUGR, TTTS | Twin 2. Epilepsy, DD, autistic tendencies, cleft lip and palate, retinopathy of prematurity | Novel deletion Chr3, supported by RNA seq data, PDCD6IP z-score = -5.71 | isomerase activity, actin binding, cell-cell adhesion, iron ion transport, homophilic cell adhesion |
| 7075 | P113 | M | 40 | Dys/SpQ | 4 | S | 7 | Cold/flu 21+ weeks | ID, postnatal seizures secondary to prenatal hypoxic ischaemic encephalopathy. Orchidopexia. | Novel deletion Chr19, supported by RNA seq data AP3D1 z-score = -5.754, LMNB2 z-score = -4.013, LSM7 z-score = -4.308, OAZ1 z-score = -7.029, PLEKHJ1 z-score = -4.035, SF3A2 z-score = -5.518, SGTA z-score = -5.045, THOP1 z-score = -5.571 | structural constituent of ribosome, negative regulation of apoptosis, translation elongation factor activity, fatty acid metabolism |
| 7132 | P440 | M | 34 | SpH |  | S | 6 |  |  | MAP7D2 (X-linked), **TBL1XR1** z-score = -4.12 | protein serine/threonine kinase activity, diacylglycerol binding, G-protein signalling, coupled to IP3 second messenger (phospholipase C activating) |
| 7133 | P441 | M | NA | H | 1 |  | 8 |  |  | Sibling with ASD | regulation of transcription, DNA-dependent, cytoskeleton organization and biogenesis, immune response |
| 7134 | P442 | M | 24 | Dys |  | S | 8 | Infection/fever during labour, history of pre-term birth | DD | Cousin with CP | histogenesis, cell adhesion, P-P-bond-hydrolysis-driven transporter activity, cell adhesion molecule activity, M phase of mitotic cell cycle |
| 7135 | P443 | F | 39 | SpH | 1 | S | 9 | Placenta praevia, bedrest from 33 weeks, postnatal psychosis |  | Maternal uncle severe physical and mental disability, possible CP, RFX2 (de novo), NR1I2 ( de novo) | nucleotide metabolism, late endosome, chromatin modification, intracellular transport |
| 7198 | P444 | F | 37 | H |  | S | 6. | Anaemia, pre-eclampsia |  |  | nuclear pore, DNA metabolism, protein-nucleus import, docking, nucleocytoplasmic transport, RNA metabolism, endomembrane system, cell cycle |
| 7199 | P445 | M | 29 | SpD | 3 | S | 9 | Alcohol 1-2 per week, IUGR |  | MYO1G (CH) | inositol/phosphatidylinositol kinase activity, RNA binding, ribonucleoprotein complex |
| 7200 | P446 | F | 27 | SpD | 2 | S | 4 | History of pre-term birth, possible incompetent cervix | Hyperbilirubinemia, hypertonia. |  | metabolism, mitotic cell cycle, response to endogenous stimulus, cell cycle |
| 7209 | P447 | M | 38 | Dys |  | S | 11 | Cold/flu 1-20 weeks, high blood pressure | Left cerebral artery infarction | Father possible mild CP, PAK3 (X-linked) | mitochondrion, DNA repair, cell cycle, main pathways of carbohydrate metabolism, oxidoreductase activity, mitotic cell cycle |
| 7210 | P448 | M | 36 | SpD | 2 |  | 12 |  |  | Great uncle CP | protein binding, intercellular junction, cell junction, porin activity |
| 7211 | P449 | F | 38 | Dys |  | S | 16 |  |  |  | structural constituent of ribosome, metal ion transport, |
| 7268 | P450 | M | 34 | D |  | S | 5 | 16 weeks – bleeding, contractions, Cold/flu, fever 21+ weeks, 33 weeks - high blood pressure, protein, decreased foetal movement, tight cord |  | ENPP4 (de novo), RAD21 (de novo), DYNC2H1 (CH), PROS1 (CH) | response to wounding, cellular defense response, transport, steroid biosynthesis |
| 7269 | P451 | F | 39 | SpD |  | S | 8 | Anaemia, tight cord, Gastric infection 21+ weeks | DD, hypoplasia of inferior cerebellar vermis. Facial glabella hemangioma | TUBA1A (de novo) | regulation of cell proliferation, phosphoric monoester hydrolase activity |
| 7270 | P452 | M | 27 | SpQ | 4 | S | 13 |  |  |  | protein-nucleus import\, docking, nucleocytoplasmic transport, RNA metabolism |
| 7315 | P114 | F | 38 | H |  | S | 2 | Tight cord around body, Gastric infection 21+ weeks | Decreased left hand movements from 2-3 months |  | enzyme linked receptor protein signaling pathway, carboxylic acid metabolism, cell surface receptor linked signal transduction, regulation of cell cycle |
| 7322 | P453 | F | 41 | SpD | 3 | S | 3 |  | Epilepsy | ZNF468 z-score = -4.33 | nucleobase, nucleoside, nucleotide and nucleic acid metabolism, ligase activity, DNA metabolism, response to DNA damage stimulus, cell cycle |
| 7323 | P454 | M | 41 | SpD | 1 | S | 3 |  |  | Cousin CP, MIIP (de novo) | signal transducer activity, receptor activity, lipid kinase activity, cell communication, cell adhesion, ion transport |
| 7370 | P455 | M | 27 | SpD | 4-5 | S | 5 | Bleeding at 27 weeks |  | PEX19 (f/shift, inherited from Father) z-score = -5.30, NDUFV3 z-score = -4.25 | regulation of apoptosis, receptor activity, cell communication, programmed cell death |
| 7371 | P456 | M | 27 | SpQ | 1 | S | 4 | Bleeding at 27 weeks, major bleeding in labour, hypothyroidism | Epilepsy, DD, hydrocephalus | LAMA3 (CH) | intracellular signaling cascade, galactosyltransferase activity, Golgi apparatus |
| 7372 | P457 | M | 40 | SpD | NA | S | 14 | Cold/flu 1-20 weeks | Borderline IQ, ASD, epilepsy. Factor V clotting problem, irregular heartbeat |  | cell communication, intracellular signaling cascade, small GTPase regulatory/interacting protein activity |
| 7385 | P458 | F | 41 | H | 1 | S | 5 | Smoking |  | FBN2 (CH) | phosphoric ester hydrolase activity, cell communication |
| 7386 | P459 | F | 30 | SpD | 2-3 | S | 4 | High blood pressure, infection/fever in labour, history pre-term birth, hypothyroidism |  | GRSF1 (f/shift, inheritance unknown) z-score = -5.10 | metal ion binding, development, transcription factor activity, cell adhesion molecule activity, skeletal development, organogenesis, cell adhesion |
| 7387 | P460 | M | 41 | H | 1 | S | 14 | Anaemia | Epilepsy, day 2 US showed global cerebral oedema | Maternal Grandmother mild one-sided weakness never diagnosed as CP, ZFYVE16 (de novo), MKI67 (CH), ZNF839 z-score = -4.74 | mitosis, microtubule cytoskeleton, motor activity, cytokinesis |
| 7388 | P461 | M | 27 | SpD | 2 | S | 8 | Asthma, cervical suture 14 weeks, fever after waters broke |  |  | cell communication, cell surface receptor linked signal transduction, actin cytoskeleton |
| 7420 | P462 | F | 40 | SpD | 4 | S | 10 | Milroy's disease (lymphodema), fever 21+ weeks, pre-term labour at 32 weeks | Holoprosencephaly, seizure disorder (thought to be neuronal migration disorder with semi-lobar holo-presencaphaly), diabetes insipidis, hearing loss, cleft lip & palate, global development delay, GORD. | Mother CP | organogenesis, development, skeletal development, defense response, response to external stimulus, enzyme linked receptor protein signaling pathway, ATP-binding cassette (ABC) transporter activity |
| 7421 | P463 | F | 37 | SpQ | 4 | S | 11 | Smoking, decreased foetal movements 32 weeks, IUGR |  | Turner syndrome, ECSIT (stop gain, inherited from Father) z-score = -4.92 | ion transport, phosphotransferase activity, alcohol group as acceptor, kinase activity |
| 7492 | P421 | F | 25 | SpQ |  | Trip | 13 | IVF | Triplet 2, non-verbal, Intrathecal Baclofen pump in situ |  | large ribosomal subunit, cell cycle, macromolecule biosynthesis, nuclear division, cell proliferation |
| 7495 | P464 | M | 30 | Dys/D | 2 | S | 6 | Placenta praevia, asthma (asthma attack at 4 months) | Severe ID, short term memory loss, severe speech delay, microcephaly. |  | sensory perception, tumor necrosis factor receptor binding, |
| 7501 | P465 | F | 34 | SpD | 3 | Tw | 4 | Vanishing twin syndrome, placental abruption due to clotting disorder, Major bleeding during labour |  | 22q duplication supported by RNA seq data | ion transporter activity, mitochondrial ribosome, transporter activity |
| 7502 | P466 | M | 37 | SpH | 1 | S | 8 | Mother and child Factor V Leiden mutation positive | Stroke at 4 years of age | Factor V Leiden mutation positive, family history epilepsy | cysteine-type endopeptidase activity, vacuole, cell communication |
| 7509 | P115 | M | 40 | SpD |  | S | 3 | Gastric infection 1-20 weeks, polyhydramnios | Moderate ID, epilepsy, hydrocephalus communicating with Blake’s pouch cyst, cerebellar folial disorganisation, incomplete bilateral hippocampal inversion, optic nerve hypoplasia, partial agenesis of corpus callosum. Adducted thumbs, severe speech delay | TUBA1A (de novo), PPT1 z-score = -4.14 | apoptosis regulator activity, cell adhesion molecule activity, apoptotic program, JNK cascade, metal ion transport, neurogenesis |
| 7510 | P116 | F | 26 | SpQ | 4 | S | 4 | Incompetent cervix, cervical cerclage, UTI, Cold/flu 1-20 weeks | ID, chronic lung disease | TTN (CH) | cell communication, signal transducer activity, signal transduction, apoptosis, cell adhesion molecule activity, calmodulin binding |
| 7511 | P117 | M | 39 | SpH | 1 | S | 4 |  | Possible seizures day 2, apnoea and desaturation. Borderline ID, epilepsy (refractory focal epileptic seizures), left homonymous hemianopia, Extensive encephalomalacia involving right frontotemporal region in keeping with previous infarct. Right brain size smaller. Cystic changes. Right lateral ventricle dilated. | CUL4B (de novo), NAA35 (de novo), NID2 (de novo), PCBP3 (de novo) | cell communication, signal transducer activity, cell-cell signaling, synaptic transmission, receptor signaling protein activity, transmission of nerve impulse |
| 7526 | P467 | F | 35 | SpD | 3 | S | 6 | Smoking | Epilepsy | TENM1 (de novo), MMADHC z-score = -3.15, ALKBH3 (f/shift, inheritance unknown) z-score = -4.43 | protein serine/threonine kinase activity, mRNA metabolism, gene silencing, spindle pole, intracellular transport |
| 7527 | P468 | F | 39 | SpD | 3 | S | 12 | Smoking | Moderate to-severe ID | SPAST (inheritance unknown), ATG13 z-score = -4.55 | nuclear division, cell cycle, DNA repair, cytokinesis, cell proliferation, nucleocytoplasmic transport |
| 7528 | P469 | F | 37 | Dysk/ Sp | 5 | S | 11 | Mother diabetes, high blood pressure, anaemia | Dystonia and athetosis | Chromosomal translocation of unknown significance, **TXN2** (splice mutation, inherited from Mother) z-score = -4.55 | aromatic amino acid family catabolism, peroxisome, oxidoreductase activity, acting on CH-OH group of donors |
| 7646 | P470 | M | 39 | H | 3 | S | 3 | Liver infection resolved after birth, dehydrated |  | **STK16** (f/shift, inheritance unknown) z-score = -4.25, | RNA modification, morphogenesis, neurogenesis, organogenesis |
| 7647 | P471 | F | 27 | SpD | 1 | S | 4 | Asthma, clots on placenta, bleeding & placental abruption |  |  | transcription, DNA-dependent, monocarboxylic acid transport, organic anion transport |
| 7648 | P472 | M | NA | AD | 2 | S | 13 | Recreational drugs | Wolff-Parkinson-White syndrome, (hypertrophic cardiomyopathy), Unilateral renal dysfunction/failure, spontaneously resolved, supraventricular tachycardia at birth |  | nuclear division, mitosis, microtubule cytoskeleton, cell proliferation, microtubule motor activity |
| 7772 | P474 | M | 40 | H | 5 | S | 3 | UTI |  |  | Function signal sequence binding, cotranslational membrane targeting, protein-ER targeting, endoplasmic reticulum |
| 7773 | P475 | M | 36 | H | 3 | S | 5 |  | Polymicrogyria, heart: abnormal arch and branching, Velo-Cardio-Facial syndrome, bilateral talipes, t-cell immunodeficency, mandibular osteomyolitis, laryngomalacia | Chr22q11 deletion supported by RNA seq | morphogenesis, GDP-dissociation inhibitor activity, cell adhesion, development, apoptosis regulator activity, organogenesis, cell communication, embryogenesis and morphogenesis, regulation of neurotransmitter levels |
| 7775 | P118 | M | 40 | H | 1 | S | 7 | Gastric infection 21+ weeks | ASD, HIE-grade II, hypoglycaemia, neonatal seizures |  | inflammatory response, innate immune response, transcription factor activity, positive regualtion of apoptosis |
| 7939 | P476 | F | 38 | SpQ | 4 | Tw | 6 | IVF twin pregnancy, TTTS, other twin did not survive | Microcephaly, bilateral closed lip schizencephaly | APC (CH), DOCK8 z-score = -4.43 | carbohydrate catabolism, glucose metabolism, energy pathways, glycolysis |
| 7957 | P119 | F | 39 | H | 2 | S | 12 |  |  |  | regulation of transcription, metabolism, development |
| 7997 | P477 | F | 40 | SpD | 4 | S | 7 |  |  |  | metabolism, phosphatidylserine metabolism, tubulin folding, transcription, DNA-dependent, response to DNA damage stimulus, receptor activity |

**Supplementary Table 1: Clinical and genetic data for cerebral palsy cases included in this study.** Abbreviations: ADHD, attention deficit hyperactivity disorder; ASD, autism spectrum disorder; Asy, asymmetric; A, ataxic; Ath, Athetosis; CH, compound heterozygous; CMV, cytomegalovirus; del, deletion; DD, developmental delay; D, diplegia; Dysk, dyskinetic; Dys, dystonic; ECG, electrocardiogram; EEG, electroencephalogram; F, female; F/shift, frameshift; GMFCS, Gross Motor Function Classification Score; GORD, Gastro-oesophageal reflux disease; H, hemiplegia; HIE, Hypoxic-ischemic encephalopathy; ID, intellectual disability; ins, insertion; IUGR, intra-uterine growth restriction; IVF, in vitro fertilisation; IVH, intraventricular haemorrhage; M, male; MCA, middle cerebral artery stroke; OCD obsessive compulsive disorder; Q, quadriplegia; S, singleton; Sp, spastic; T, triplegia; Trip, triplet; Tw, twin; TTTS, twin-twin transfusion syndrome; US, ultrasound; UTI, urinary tract infection.
